# Exploring the crop epigenome: a comparison of DNA methylation profiling techniques

**DOI:** 10.3389/fpls.2023.1181039

**Published:** 2023-05-30

**Authors:** Dolores Rita Agius, Aliki Kapazoglou, Evangelia Avramidou, Miroslav Baranek, Elena Carneros, Elena Caro, Stefano Castiglione, Angela Cicatelli, Aleksandra Radanovic, Jean-Paul Ebejer, Daniel Gackowski, Francesco Guarino, Andrea Gulyás, Norbert Hidvégi, Hans Hoenicka, Vera Inácio, Frank Johannes, Erna Karalija, Michal Lieberman-Lazarovich, Federico Martinelli, Stéphane Maury, Velimir Mladenov, Leonor Morais-Cecílio, Ales Pecinka, Eleni Tani, Pilar S. Testillano, Dimitar Todorov, Luis Valledor, Valya Vassileva

**Affiliations:** ^1^ Centre of Molecular Medicine and Biobanking, University of Malta, Msida, Malta; ^2^ Biology Department, Ġ.F.Abela Junior College, Msida, Malta; ^3^ Department of Vitis, Institute of Olive Tree, Subtropical Crops and Viticulture (IOSV), Hellenic Agricultural Organization-DIMITRA (ELGO-DIMITRA), Athens, Greece; ^4^ Laboratory of Forest Genetics and Biotechnology, Institute of Mediterranean Forest Ecosystems, Hellenic Agricultural Organization-DIMITRA (ELGO-DIMITRA), Athens, Greece; ^5^ Mendeleum-Insitute of Genetics, Faculty of Horticulture, Mendel University in Brno, Lednice, Czechia; ^6^ Center for Biological Research (CIB) of the Spanish National Research Council (CSIC), Madrid, Spain; ^7^ Centro de Biotecnología y Genómica de Plantas, Instituto Nacional de Investigación y Tecnología Agraria y Alimentaria (INIA), Universidad Politécnica de Madrid (UPM), Madrid, Spain; ^8^ Department of Chemistry and Biology ‘A. Zambelli’, University of Salerno, Fisciano, Italy; ^9^ Institute of Field and Vegetable Crops, National Institute of Republic of Serbia, Novi Sad, Serbia; ^10^ Department of Clinical Biochemistry, Faculty of Pharmacy, Collegium Medicum in Bydgoszcz, Nicolaus Copernicus University in Toruń, Bydgoszcz, Poland; ^11^ Centre for Agricultural Genomics and Biotechnology, Faculty of Agricultural and Food Sciences and Environmental Management, University of Debrecen, Nyíregyháza, Hungary; ^12^ Genomic Research Department, Thünen Institute of Forest Genetics, Grosshansdorf, Germany; ^13^ BioISI – BioSystems & Integrative Sciences Institute, Faculdade de Ciências, Universidade de Lisboa, Lisbon, Portugal; ^14^ Plant Epigenomics, Technical University of Munich (TUM), Freising, Germany; ^15^ Faculty of Science, University of Sarajevo, Sarajevo, Bosnia and Herzegovina; ^16^ Department of Vegetables and Field Crops, Agricultural Research Organization, Volcani Center, Institute of Plant Sciences, Rishon LeZion, Israel; ^17^ Department of Biology, University of Florence, Sesto Fiorentino, Italy; ^18^ Laboratoire de Biologie des Ligneux et des Grandes Cultures EA1207 USC1328, INRAE, Université d’Orléans, Orléans, France; ^19^ Faculty of Agriculture, University of Novi Sad, Novi Sad, Serbia; ^20^ Linking Landscape, Environment, Agriculture and Food (LEAF), Institute of Agronomy, University of Lisbon, Lisbon, Portugal; ^21^ Centre of Plant Structural and Functional Genomics, Institute of Experimental Botany of the Czech Academy of Sciences, Olomouc, Czechia; ^22^ Laboratory of Plant Breeding and Biometry, Department of Crop Science, Agricultural University of Athens, Athens, Greece; ^23^ Department of Molecular Biology and Genetics, Institute of Plant Physiology and Genetics, Bulgarian Academy of Sciences, Sofia, Bulgaria; ^24^ Plant Physiology, Department of Organisms and Systems Biology and University Institute of Biotechnology of Asturias, University of Oviedo, Oviedo, Spain

**Keywords:** crop epigenome, DNA methylation profiling, bisulfite sequencing, next-generation sequencing, immunological techniques, mass spectrometry, DNA methylation modulation

## Abstract

Epigenetic modifications play a vital role in the preservation of genome integrity and in the regulation of gene expression. DNA methylation, one of the key mechanisms of epigenetic control, impacts growth, development, stress response and adaptability of all organisms, including plants. The detection of DNA methylation marks is crucial for understanding the mechanisms underlying these processes and for developing strategies to improve productivity and stress resistance of crop plants. There are different methods for detecting plant DNA methylation, such as bisulfite sequencing, methylation-sensitive amplified polymorphism, genome-wide DNA methylation analysis, methylated DNA immunoprecipitation sequencing, reduced representation bisulfite sequencing, MS and immuno-based techniques. These profiling approaches vary in many aspects, including DNA input, resolution, genomic region coverage, and bioinformatics analysis. Selecting an appropriate methylation screening approach requires an understanding of all these techniques. This review provides an overview of DNA methylation profiling methods in crop plants, along with comparisons of the efficacy of these techniques between model and crop plants. The strengths and limitations of each methodological approach are outlined, and the importance of considering both technical and biological factors are highlighted. Additionally, methods for modulating DNA methylation in model and crop species are presented. Overall, this review will assist scientists in making informed decisions when selecting an appropriate DNA methylation profiling method.

## Introduction

**Figure 1:**
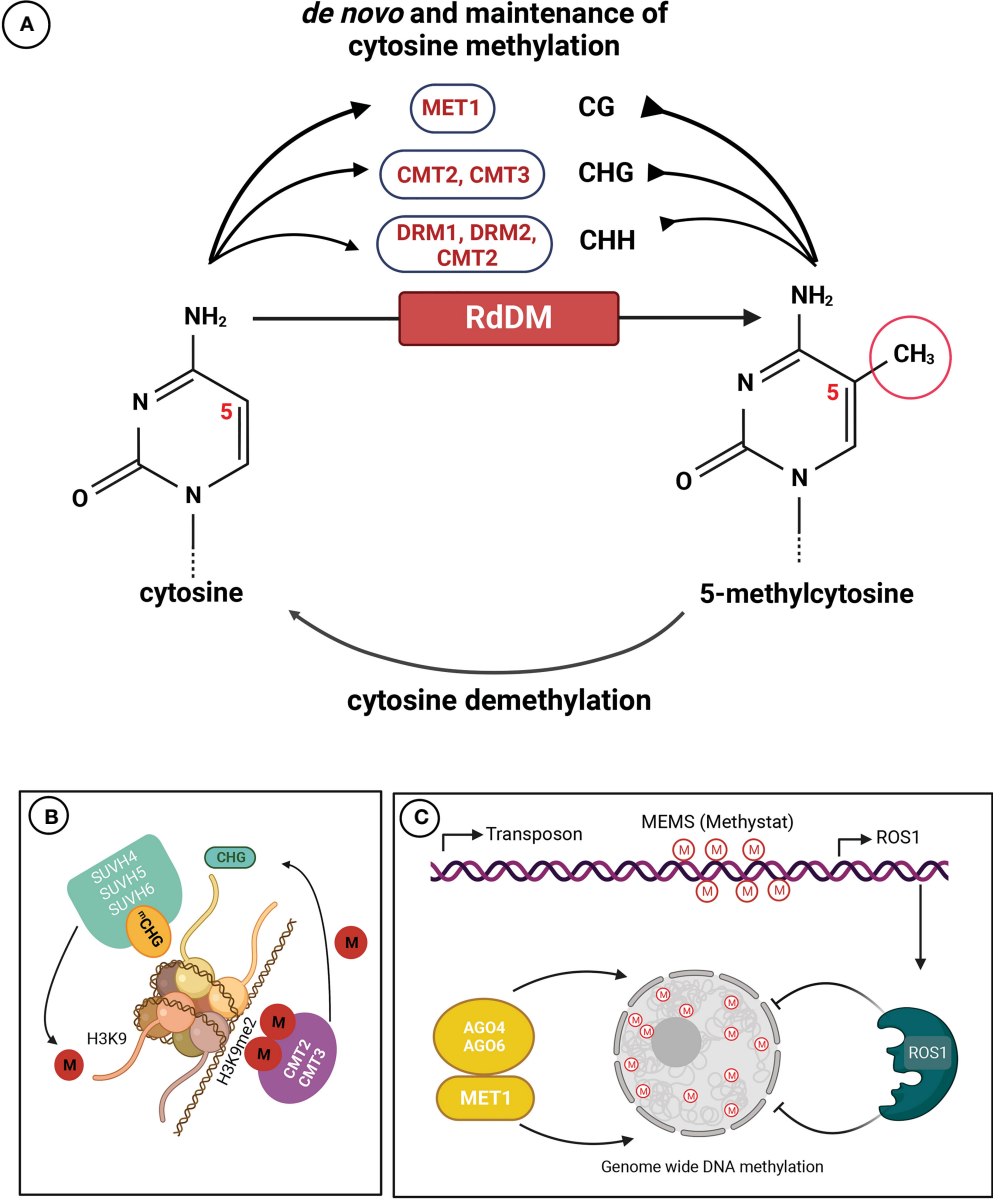
Mechanisms of DNA cytosine methylation in plants. **(A)** Maintenance and *de novo* DNA methylation occur in all sequence contexts (CG, CHG, and CHH; where H = A, C, or T). Methyltransferase 1 (MET1) maintains methylation in the CG context, CHROMOMETHYLASE 2 (CMT2) or CMT3 catalyzes and maintains methylation in the CHG context, DOMAINS REARRANGED METHYLASE 2 (DRM2) and CMT2 accomplish and maintain methylation in the CHH context. The RdDM pathway conducts *de novo* DNA methylation in all sequence contexts. **(B)** The attraction of histone H3 lysine 9 (H3K9)-specific suppressor of variegation 3-9 homolog proteins (SUVH4, SUVH5 and SUVH6) results in the formation of dimethylated H3K9 (H3K9me2), and the recruitment of CMT2 and CMT3, creating a self-reinforcing feedback loop. **(C)** Methylation of the methylation monitoring sequence (MEMS) in the promoter region of the Repressor of silencing 1 (ROS1) gene is crucial for its transcription. Cytosine methylation at MEMS is regulated by both MET1/RdDM and ROS1, enabling sensing/monitoring of methylation levels and maintaining DNA (de)methylation homeostasis. The methyl group is denoted by “M”, and methylation state is represented as “me/m” (Adapted from Kumar and Mohapatra, 2021).

Since the introduction of the term by Waddington (1942), the definition of epigenetics has evolved over time. The latest definition describes it as a genetic subfield dealing with mitotically and/or meiotically heritable changes in gene expression patterns that occur without alterations in DNA sequence (Deans and Maggert, 2015). Epigenetics focuses on studying the chemical changes in chromatin that are often referred to as ‘epigenetic marks’. One of the most widespread epigenetic marks is cytosine methylation, which is catalyzed by different enzymatic pathways (**Figure 1**).

In plants, cytosine methylation occurs at symmetric CG, but also at symmetric CHG and asymmetric CHH sites, where H is any nucleotide except G (**Figure 1A**). *De novo* methylation in CG, CHG and CHH contexts is established mainly by DOMAINS REARRANGED METHYLTRANSFERASE1 (DRM1) and 2 (DRM2) (Cao and Jacobsen, 2002; Law and Jacobsen, 2010; He et al., 2014), and directed by small interfering RNAs (siRNAs) (Pikaard and Scheid, 2014). Methylation maintenance is carried out through the activity of METHYLTRANSFERASE 1 (MET1) assisted by VIM (VARIANT IN METHYLATION) family proteins (VIM1-VIM3) that preferentially maintains the CG site methylation. MET1 recognizes hemimethylated CG dinucleotides following DNA replication and methylates the cytosine in the daughter strand (Finnegan and Dennis, 1993; Kankel et al., 2003), CHROMOMETHYLASE 2 (CMT2) and 3 (CMT3) maintain non-CG methylation (Stroud et al., 2013), with CMT2 linked to the asymmetric CHH methylation of constitutive heterochromatin, whereas CMT3 is more closely associated with CHG methylation (Ning et al., 2020). DRM1 and DRM2 contribute to asymmetric CHH methylation in euchromatin and at the edge of long transposable elements (TEs) through RNA-directed DNA methylation (RdDM), which involves siRNAs and scaffold RNAs in addition to an array of proteins (Zemach et al., 2013; He et al., 2021). Heterochromatic regions are densely populated by TEs and repetitive sequences and feature dense cytosine methylation in all three sequence contexts (Ashapkin et al., 2020). Heavy methylation of these regions ensures faithful silencing and prevents the formation of aberrant structural variations through TE mobilization or unequal crossing-over at meiosis (Underwood et al., 2017; Lee et al., 2020). Protein-coding genes are broadly divided into three groups: gene body methylated (gbM) genes enriched for CG methylation and depleted for non-CG methylation; TE-like genes enriched for methylation in the three contexts, and unmethylated genes lacking cytosine methylation (Schmitz et al., 2019; Crisp et al., 2020; Muyle et al., 2022).

In general, TE-like genes tend to be transcriptionally silenced by cytosine methylation, whereas gbM genes display intermediate expression levels that are not clearly linked to cytosine methylation (Schmitz and Bewik, 2017). Although gene promoters are typically unmethylated, there are examples in crops when promoter hypermethylation tends to inhibit transcription (Liu et al., 2017; Lucibelli et al., 2022). Hypermethylated promoters often coincide with gene-proximal TE insertions, whose methylated status establishes a repressive chromatin environments that are incompatible with active transcription (Slotkin and Martienssen, 2007). Spreading of cytosine methylation from gene proximal TEs can methylate cytosine nucleotides within transcription factor binding motifs, thus reducing binding affinity. However, a subclass of transcription factors displays an increased binding affinity for methylated motifs; in this case, the hypermethylation of promoters can stimulate transcription (Bartlett et al., 2017). Methylation of introns can impact the production of alternative versions of a gene RNA, known as transcript variants, occurring through a process called polyadenylation, where specific regions of RNA are modified (Saze et al., 2013; Wang et al., 2013).

Active demethylation of 5-methylcytosine (5mC) in plants is initiated by DNA demethylases and takes place *via* a base-excision-repair pathway involving DNA glycosylases (Zhu, 2009). In *Arabidopsis thaliana*, researchers have identfied a family of four bifunctional 5mC DNA glycosylases, including REPRESSOR OF SILENCING 1 (ROS1), TRANSCRIPTIONAL ACTIVATOR DEMETER (DME), DEMETER-LIKE PROTEIN 2 (DML2) and 3 (DML3) (Gong et al., 2002; Ortega-Galisteo et al., 2008), which can excise 5mC from all cytosine sequence contexts (Agius et al., 2006; Penterman et al., 2007; Zhu et al., 2007). Passive demethylation can also occur after DNA replication if maintenance DNA methyltransferases are inhibited or absent (Wu and Zhang, 2010).

The significance of cytosine methylation for plant evolution is associated not only with the regulation of gene expression but also with silencing and reactivation of TEs (Madlung et al., 2005; Miryeganeh and Saze, 2020). On a whole genome level, CHH methylation is relatively conserved across plant species, whereas CHG methylation varies and is often linked to genome size, confirming that TE amplifications depend on non-CG methylation (Takuno et al., 2016; Vidalis et al., 2016). Cytosine methylation is crucial for many developmental cues and processes, such as flowering, senescence time, gametogenesis, imprinting and memory of parental origin (Miryeganeh and Saze, 2020). Silencing sperm and ovule alleles either maternally or paternally ensures epigenetic memory of gene expression in double fertilization, leading to differential expression of maternal and paternal alleles in the endosperm (Iwasaki and Paszkowski, 2014). DNA methylation levels vary with organs within the same individual, e.g. tomato (*Solanum lycopersicum*) leaves display an average level of about 22.08%, lower than fruits (24.33%) (Zhong et al., 2013), while the average level in *Fragaria* × *ananassa* leaves is slightly higher than in immature fruits (Cheng et al., 2018).

Plant plasticity under changing environment can be linked to epigenetic modifications often leading to heritable “epialleles” or “epimutations”. Experiments with Arabidopsis inbred lines of mosaic epigenome (epigenetic recombinant inbred lines, epiIRLs) show that DNA methylation can affect plant plasticity including stress resilience (Cortijo et al., 2014; Kooke et al., 2015; Latzel et al., 2016). Epigenetic variation in natural populations corresponds with phenotypic differentiation (Gáspár et al., 2019), contributing to better adaption of the plant to specific environments (Boquete et al., 2021). Epigenetic marks and their changes upon environmental cues and stress can be transmitted to progeny resulting in phenotypic variation that can increase population long-term survival, particularly in clonally reproducing plants (Allendorf, 2017). Some epigenetic marks are lost during gametogenesis, limiting the heritability of stress-induced epigenetic marks, while others are added *de novo* (epigenetic reprogramming) (Boquete et al., 2021). DNA methylation reinforcements through the activities of MET1 (CG methylation), CMTs (CHG and CHH methylation), and RNA-directed DNA methylation (*de novo* methylation) can contribute to transgenerational transmission through self-reinforcing loops (Erdmann and Picard, 2020).

As demonstrated above, cytosine methylation regulates gene expression and various biological processes. Yet, the methods to study methylation patterns are very diverse and have greatly evolved in recent years. Early chromatography techniques have been replaced by advanced methods for genome-wide methylation profiling at single-nucleotide resolution, facilitated by next-generation sequencing (NGS) and sequencing-based DNA methylation mapping (Kuo et al., 1980; Harrison and Parle-McDermott, 2011). This review looks at the commonly used technologies for analysing DNA methylation in crops, discusses the advantages and limitations of each methodological approach, and highlights the importance of considering both technical and biological factors when selecting a method.

## Methodologies for measuring DNA methylation

### Methylation sensitive amplification polymorphism technique

Methylation Sensitive Amplified Polymorphism (MSAP) has been widely used over the past three decades for assessing DNA methylation changes in a range of model and non-model plants. Essentially, MSAP is a modification of the amplified fragment length polymorphism (AFLP) technique, originally described by Vos et al. (1995), except that the frequent cutter enzyme MseI is substituted by the methylation-sensitive isoschizomers HpaII and MspI. Hence, the MSAP method utilizes HpaII and MspI, which recognize the same target site (5’-CCGG-3’). However, their ability to cleave is based on the methylation state of specific cytosine residues in the sequence. Specifically, HpaII only cleaves sites with hemimethylated external cytosines (^m^CCGG), whereas MspI cleaves at hemi- or fully methylated internal cytosines (C^m^CGG). None of the enzymes cleave sites that are fully methylated at the external cytosine or hemi- or fully methylated internal and external cytosines. Conversely, both enzymes can digest unmethylated ‘5-CCGG-3’ sequences. These enzymes are combined with EcoRI, which is marginally affected by cytosine methylation. EcoRI/HpaII and EcoRI/MspI DNA digests are ligated to specific adapters and ligated fragments undergo rounds of preselective and selective PCR amplification. Selective amplification with specific fluorescently labelled primers produces PCR fragments that are resolved on capillary electrophoresis detection systems. Raw data matrix of presence and absence of fragments are translated into a binary character matrix (0, absence; 1, presence). The binary information of each fragment is associated with its methylation status (Schulz et al., 2013).

MSAP approach was first utilized to determine DNA methylation patterns during fungal development (Reyna-López et al., 1997). It was subsequently modified for a diversity of model and non-model plant species to detect methylation patterns associated with plant growth and development (different developmental stages, tissues, organs, abiotic stress responses, grafted plants, tissue culture, inter- and intra-population variability at different environments).

Due to space limitations and a plethora of reports (over 100 MSAP studies in plants), only a few examples are provided. MSAP has detected significant DNA methylation changes under drought stress in *Oryza sativa* (rice) (Wang et al., 2011), *Lolium perenne* (Tang et al., 2014a), *Hordeum vulgare* (barley) (Chwialkowska et al., 2016), *Vicia faba* (Abid et al., 2017), and recurrent water deficit in *Medicago sativa* (Ventouris et al., 2020). Similarly, altered DNA methylation patterns have been observed in response to salt stress in *Gossypium hirsutum* and *Brassica napus* (rapeseed) (Marconi et al., 2013; Wang et al., 2016), chilling stress in *Malus × domestica* (Kumar et al., 2016), laser radiation stress in rice (Li et al., 2017), and aluminum stress in triticale (Bednarek et al., 2017). Further, differential methylation states are established in grapevine under UV-B radiation, water deficit, and ABA exposure (Marfil et al., 2019). Recently, MSAP profiling has detected global methylation changes in greenhouse rocket at varying root-zone temperatures (Tsaballa et al., 2022), and in rice cultivars under high temperatures (Li et al., 2022). DNA methylation has been evaluated in scions of inter-species grafting of *Solanaceae* (Wu et al., 2013), inter- and intra-species grafting of *Cucurbitaceae* (Avramidou et al., 2014; Xanthopoulou et al., 2019), and heterografts of *Hevea brasiliensis* (Uthup et al., 2018). MSAP has detected adaptive epigenetic differentiation in mangrove populations grown in contrasting environments (Lira-Medeiros et al., 2010), in *Hydrocotyle vulgaris* populations under different flooding regimes (Wang et al., 2022), and in grapevine clones or varieties grown in diverse geographical locations (Xie et al., 2017; Baránková et al., 2021; Varela et al., 2021). It has been utilized to investigate the association of DNA methylation with phenotypic variance in maize (*Zea mays*) (Xu et al., 2019), and in populations of non-model plants, including the perennial herb *Scabiosa columbaria* (Groot et al., 2018), the ornamental tree *Prunus mume* (Ma et al., 2018), cork oak (*Quercus suber*) (Inácio et al., 2017) and *Vitex negunda* (Lele et al., 2018). Additionally, MSAP is used to evaluate conservation strategies of genetic material, such as cryopreservation and *in vitro* plant conservation (Ibáñez et al., 2019; González-Benito et al., 2020).

The major advantage of the MSAP technique is its wide feasibility across all species regardless of reference genome availability, cost-efficiency independently of genome size and complexity, and suitability for profiling large sample sets. MSAP has also some limitations. It detects DNA methylation patterns at anonymous loci randomly distributed throughout the genome and cannot provide information on specific genes or genomic regions. In some studies differentially methylated fragments were extracted from polyacrylamide gels, sequenced and identified through BLAST homology searches to overcome this limitation (Wang et al., 2011; Cicatelli et al., 2014; Wang et al., 2015a); a rather laborious method that provides limited information due to many small-sized bands. MSAP also cannot detect cytosine methylation at CHH sites (important for gene and transposon regulation), as HpaII and MspI enzymes recognize only ‘CCGG’ sites within CG or CHG sequence contexts. Additionally, scoring of fragment methylation status and interpretations may vary among labs (Fulneček and Kovařík, 2014). The most common scoring indicates four conditions: Condition I, “CCGG”, unmethylated/presence of a band for both EcoRI/HpaII and EcoRI/MspI digests (H1, M1); Condition II, “C^m^CGG”-hemi or full methylation of internal cytosine/presence of band only for the EcoRI/MspI digest (H0, M1); Condition III, “^m^CCGG”-hemimethylation of external cytosine/presence of band only for the EcoRI/HpaII digest (H1, M0), and Condition IV, “^m^C^m^CGG”-hemi or full methylation of both cytosines or full methylation of external cytosines/absence of band for both EcoRI/HpaII and EcoRI/MspI digests (H0, M0). However, Condition IV is considered full methylation by some researchers but uninformative by others. A mutation in the CCGG sequence may hinder digestion and affect conclusions. To ensure accurate inference of global DNA methylation percentages and facilitate cross-laboratory comparisons, a consistent protocol for scoring and interpreting matrix data is crucial. Schulz et al. (2013) proposed an R-based environment for population studies to address these issues. They highlighted the importance of a holistic approach for scoring in population studies (Avramidou et al., 2015; Avramidou et al., 2021), as demonstrated in their analysis of Prunus epigenome.

Overall, MSAP remains a valuable low-cost tool for assessing DNA methylation, making it a popular choice in many laboratories. A potential limitation of this technique is related to the detection of changes specifically in CCGG sequences recognized by highly-sensitive restriction enzymes, which limits detection of other types of methylation. Increasing availability of model and non-model species genomes, along with advances in NGS technology, has paved the way to the elaborate technique of MSAP-seq coupling classical MSAP and high throughput sequencing.

### МSAP linked to NGS technologies

Original MSAP methodology has a major limitation regarding the lack of knowledge of DNA traits with different methylation status. To overcome this limitation, a modified MSAP protocol, replacing the conventional separation of amplicons on polyacrylamide gels with direct high-throughput NGS sequencing, followed by an automated data analysis, has been proposed (Baránek et al., 2016; Chwialkowska et al., 2017; Chwialkowska et al., 2019; Guarino et al., 2020). This technique include: 1) parallel and double enzymatic digestions of genomic DNA with two different pairs of endonucleases (EcoRI with MspI or HpaII) to obtain a pool of DNA fragments; 2) ligation of DNA fragments to specific adapters flanking the restriction site; 3) PCR amplification to obtain a representative pool of DNA fragments; 4) NGS library preparation for amplicon analysis and sequencing, performed differently based on the technology, chemistry, detection system, and method of amplification in different generations of sequencing platforms; and 5) data analysis. Nonetheless, some steps of each protocol are modified and report different NGS data analyses through specific bioinformatic tools. Baránek et al. (2016) first used a standard MSAP analysis followed by deep amplicon sequencing with NGS technology. Sequence quality was verified, and low-quality reads were bioinformatically excluded. After adaptor trimming, contigs were assembled using Geneious 8 software and compared by blasting.

Chwialkowska et al. (2017) introduced the MSAP-Seq method, which employs an automatic pipeline called MSEQER. After purifying and fragmenting amplicons by sonication, short tags are created for easy library preparation and high-throughput sequencing. Dedicated MSEQER software is used for automated MSAP-Seq data analysis, including mapping preprocessed reads to an appropriate reference genome for identifying specific genomic sequences. Deep sequencing of MSAP-Seq amplicons allows for quantitative characterization of observed DNA methylation changes through the evaluation of fold change values of the abundance of normalized reads. Guarino et al. (2020) propose MSAP-NGS coupled technology, which reduces PCR amplification steps and applies appropriate biostatistical analysis of NGS data, especially for plant species with unsequenced genomes. After sequence quality test and adaptor trimming, all reads are used to assemble a reference genome *de novo*. The contigs obtained by read mapping on the assembled genome can be compared. To identify specific genomic sequences, contigs are mapped to an appropriate reference genome. Comparing the fragments derived from both digestion patterns within each single sample and among all the analyzed ones allows to identify genes not-affected or affected by DNA methylation. This can include double-strand methylation of inner cytosine, hemimethylation of inner cytosine, or hemimethylation of CHG-sites (M0-H1). The overall experimental pipelines and approaches for sequence analysis are summarized in **Supplementary Table 1**.

Baránek et al. (2016) used MSAP standard analysis followed by NGS of PCR selective amplicons to study epigenetically-induced changes in two wheat (*Triticum aestivum*) genotypes and their somaclones with changed heritable phenotypes linked to breeding value, i.e., improved lodging resistance and grain yield. Over 100 differentially methylated amplicons are identified, highlighting the crucial role of methylation in the activation/deactivation of TEs and the short-term and long-term dynamics of plant genomes. MSAP-Seq method (Chwialkowska et al., 2017) has been validated in barley exposed to stress. A first case study of the leaf methylome in plants grown under dehydration and rewatering allowed identification of ∼3000 sites with methylation changes under drought, many located in genes or repetitive elements. The authors also compared the methylomes of barley organs (leaf vs. root) under drought and rewatering, validating the MSAP-Seq method for this kind of analysis. Interestingly, under stress, some gene regions underwent transient and reversible methylome modifications, while many repetitive elements underwent irreversible methylation or reversible demethylation.

Guarino et al. (2020) used MSAP-NGS to explore DNA methylation in white poplar monoclonal stands from Malta, investigating if epigenetic biodiversity enhances plant adaptation to diverse pedoclimatic conditions. They assemble a high-quality *de novo* reference genome for *Populus alba* from NGS data and identified genes affected by DNA methylation by comparing amplicons from different digestion patterns within and among samples. They also analyzed DNA methylation status in each sample to discover pathways enriched with genes having varying DNA methylation levels and to identify potential DNA sequences involved in epigenetically-driven processes in white poplar.

The combination of the standard MSAP technique with NGS offers undoubtedly more advantages than limitations. The main benefits include: i) applicability to species with large and complex genomes or low gene content, as well as those with unannotated genomes; ii) the ability to target many different genomic sites, including gene-rich genomic regions, and to analyze DNA methylation in hundreds of thousands of sites across the genome (Ashikawa, 2001); iii) its relatively easy and cost effective implementation; and iv) the availability of different pipelines for bioinformatic analysis. The only limitation is detection of changes solely in CCGG sequences recognized by sensitive restriction enzymes, which limits detection of other methylation types. Similar to the standard MSAP technique, this method is unable to recognize methylation events outside of the specific sequence CCGG, or to overcome other limitations resulting from the use of the same restriction enzymes.

### Bisulfite sequencing-based methods

#### Locus specific bisulfite sequencing

**Figure 2:**
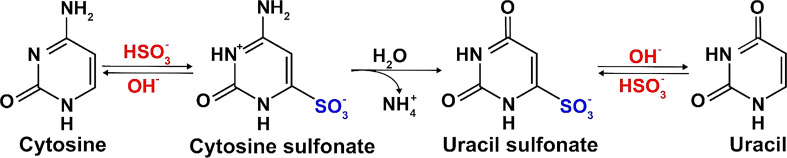
Principle of the conversion of cytosine to uracil by bisulfite-treatment.

This method is based on sodium bisulfite-mediated conversion of cytosines to uracils in single-stranded DNA (**Figure 2**), followed by PCR amplification of specific loci within the modified DNA, their cloning and sequencing by Sanger method. Sodium bisulfite deamination reactivity discriminates between cytosine and 5mC. Cytosine is deaminated to uracil, but this reaction is blocked by cytosine methylation at the 5-carbon position. Subsequent PCR using the bisulfite-treated DNA as a template leads to uracils being amplified as thymines, whereas methylated cytosines remain as cytosines (Frommer et al., 1992; Clark et al., 1994). Cloning, sequencing and comparing of the amplified DNA to the reference genome sequence can then be used to draw an exact methylation map from individual DNA molecules and score the frequency with which cytosine residues are methylated in the original DNA sample.

Sodium bisulfite conversion of genomic DNA, first described in the 90’s (Frommer et al., 1992; Clark et al., 1994), is considered the gold standard technology for studying DNA methylation. It allows for identification of 5mC at a single base-pair resolution and has been applied to DNA from many organisms, including plants.

Foerster and Scheid (2010) describe a detailed protocol for specific loci bisulfite sequencing (BS), however, here we present the most important steps and considerations on how to optimize it.

*Genomic DNA extraction*. The starting genomic DNA needs to be clean and intact (high molecular weight) to give trustworthy results. After extraction, the samples should be subjected to RNase A and even Proteinase K treatment if protein contamination is suspected, and then quantified.

*Sodium bisulfite conversion*. Most commercially available bisulfite conversion kits are applicable to very small amounts of DNA and guarantee optimal bisulfite conversion rates and DNA integrity, since they include reagents minimizing the depurination caused by harsh conversion conditions (Kint et al., 2018). If problems are observed, it is advisable to increase the denaturalization incubation time to guarantee access of bisulfite to single-stranded DNA.

*PCR*. To amplify the locus of interest accurately, primer design is crucial. Henderson et al. (2010) provides comprehensive coverage of this critical step.

Firstly, sodium bisulfite treatment damages DNA, making it difficult to amplify products over 500 bp.

Thus, to avoid amplifying undamaged, unconverted longer fragments, primer design should target 250-300 bp products. Another strategy to minimize unconverted DNA amplification is to design primers biased to amplify fully converted DNA molecules. All cytosines in the primer should be changed to thymine, except those in CG, which are methylated in a high percentage, and thus, should be changed to Y (C or T). The number of degenerate positions in the primer should be kept small, less than 3. Primers should end with one or multiple cytosines in the CHH context, changed to thymine in the primer, since they are rarely methylated. For high annealing temperature of primers without increasing their length, it is recommended to select a relatively G-rich region, as unmethylated cytosines will be converted to uracil. The primer length should be adjusted to achieve an annealing temperature above 65°C with no more than a 4°C difference between them. It is advisable to run an initial gradient PCR with new bisulfite primers to determine the optimal amplification conditions. Like all PCR reactions, primers for bisulfite-treated DNA amplification should avoid secondary structures, dinucleotide repeats, stretches of the same base longer than four, and regions with homology outside of the target. There are publicly available programs, such as BisPrimer and Kismeth for user-friendly primer design for bisulfite-treated DNA amplification in angiosperms (Gruntman et al., 2008; Kovacova and Janousek, 2012). As previously mentioned, when amplifying fragmented bisulfite-treated DNA, it may impede DNA amplification. If the first PCR fails, consider designing nested primers for a second PCR using 1 µl of the first PCR product as a template.

*Cloning and sequencing*. To confirm the expected size of the PCR product, gel electrophoresis analysis is performed. The product is purified from the gel to remove primer dimers, then cloned into a vector, and after bacterial transformation, DNA from at least 20 independent colonies should be sequenced.

*Data analysis*. Once the sequencing data are obtained, it is important to consider that each file should represent an independent DNA molecule. Sibling clones with identical patterns of methylation should be eliminated and only one should be included for analysis. Given the low frequency of methylation at CHH sites, it is unlikely for two independent clones to possess identical methylation patterns. Next, individual sequencing files are aligned and compared with the reference, which can be challenging due to different reading starts and sequence heterogeneity after conversion. There are several publicly available software tools, like Kismeth (Gruntman et al., 2008) and Cymate (Hetzl et al., 2007), that support bisulfite data analysis using algorithms that consider plant-specific DNA methylation features.

As stated above, bisulfite DNA conversion provides single base-pair resolution of methylation patterns making it a valuable tool for analyzing methylation in different cytosine contexts. Methylation levels in different contexts vary (Cokus et al., 2008) and are maintained by different mechanisms (Zhang et al., 2018a).

Sodium bisulfite can discriminate between methylated/unmethylated cytosines with high reproducibility at high temperature and low pH. However, hard conversion conditions can cause DNA fragmentation and issues in PCR amplification. Achieving a balance between conversion efficiency and DNA integrity is crucial. Mild denaturation and conversion conditions can result in unconverted genomic DNA being cloned and sequenced, which can appear as clones with many adjacent “methylated” cytosines in all contexts. To ensure complete conversion, an unmethylated genomic target region can be analyzed. For non-model organisms where this information is not available, exogenous unmethylated DNA can be added to the study sample to check later for complete conversion (Foerster and Scheid, 2010). As have already been discussed, designing primers to amplify converted DNA can be challenging without prior knowledge of the methylation degree of a specific region under certain conditions. A single primer pair permits analysis of one DNA strand through hairpin-bisulfite strategies that allow the analysis of both strands simultaneously to measure the extent of methylation symmetry between the complementary strands of individual DNA molecules (Laird et al., 2004). Although high-resolution nucleotide data are provided, the information is limited to 200-500 bp at a specific genomic locus. Combining bisulfite conversion with other sequencing strategies can provide access to larger amounts of information, which will be discussed in the next section.

#### Whole genome bisulfite sequencing

Genome-wide DNA methylation analysis is extensively used for genome characterization and evaluation of differential DNA methylation (Beck et al., 2022). Bisulfite DNA sequencing has been introduced by Frommer et al. (1992) and paved the way for NGS technique called whole-genome bisulfite sequencing (WGBS), which enables high-throughput analysis of DNA methylation. WGBS involves three main steps: 1) library preparation, 2) sequencing, and 3) alignment and quality control (Gouil and Keniry, 2019). An important step in preparing a WGBS library is the bisulfite conversion of unmethylated cytosine to uracil (**Figure 2**). The conversion includes hydrolytic deamination of cytosine sulfonate to uracil sulfonate, followed by desulfonation to uracil. The treated dsDNA is sequenced using NGS, and then PCR translates uracil into thymine. This base pair shift causes cytosine/thymine polymorphism, which is quantified, visualized, and compared to specific sites through the comparison of reads with the original strand or a reference genome (Grehl et al., 2018). WGBS library preparation consists of attaching adapters to a pool of DNA fragments (Head et al., 2014). The workflow involves three key steps: 1) sodium bisulfite treatment conversion, 2) adapter attachment to the fragment, and 3) sequencing library amplification using PCR-based methods. Depending on the priority of adapter ligation and indexing, library preparation methods are categorized as pre-bisulfite or post-bisulfite. Several WGBS library comparisons have been conducted based on datasets, protocols, quantification, and interpretation of methylation data (Zhou et al., 2019; Han et al., 2022). Bisulfite conversion causes DNA degradation of up to 90% of the DNA input (Grunau et al., 2001; Holmes et al., 2014). To reduce noise (bias) in WGBS libraries caused by bisulfite conversion, Olova et al. (2018) recommend filtering reads with three or more consecutive unconverted CH cytosines, even in datasets with high overall conversion rates. Researchers can also work with amplification-free libraries to avoid amplification-related bias (McInroy et al., 2016). Examples for library preparation methods with pre-bisulfite strategy are alkaline, heat, KAPA (heat and alkaline), and Am-BS; while methods with post-bisulfite strategy are PBAT (heat), ampPBAT (heat and alkaline), and EpiGnome (heat) (Olova et al., 2018).

**Figure 3:**
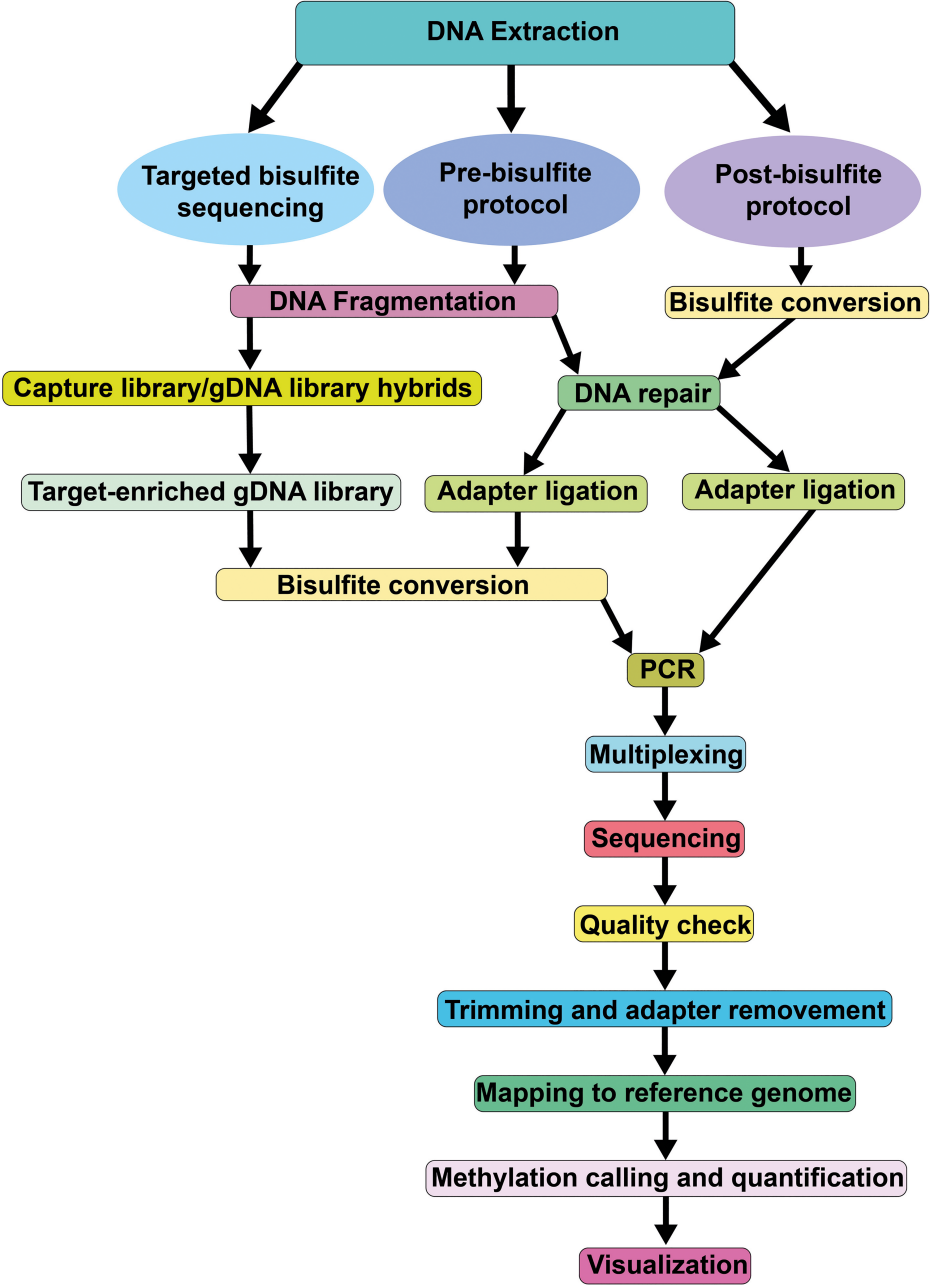
Pipeline of the Whole Genome Bisulfite Sequencing method.

Processing and analyzing WGBS datasets is computationally demanding, requiring significant memory and storage resources. **Figure 3** summarizes the pipelines and components for evaluating WGBS datasets. In a recent benchmark study with 14 alignment algorithms for WGBS in mammals, Gong et al. (2022) documented that Bwa-meth, BSBolt, BSMAP, Bismark-bwt2-e2e and Walt exhibited higher uniquely mapped reads, mapped precision, recall, and F1 score than others. However, performance statistics for mammalian genomes may not be directly applicable to crop genomes, which are typically more complex. Since most algorithms perform three letter alignments by converting cytosines in reads and in the reference genome to “T”, alignment rates for WGBS are much lower than for DNA-seq data. In streamlined genomes like *Arabidopsis*, 150 bp pair-end reads achieve alignment rates of ~70%, while complex crop genomes, such as maize, have rates of only 20-30%. Third generation long-read sequencing technologies partly overcome this limitation (discussed below). After mapping the reads to the reference genome, the methylation level of each cytosine needs to be quantified. This is typically done by calculating the ratio of methylated reads to total reads at that position. In plant genomes, methylation levels for CG context cytosines are either close to 0 or 1, while non-CG context cytosines, particularly CHH, have more variability and a narrower dynamic range (e.g. 0 to 0.4). This is probably linked to the fact that this context is more susceptible to tissue and/or cellular heterogeneity, combined with the fact that methylation in CHH is more dynamically maintained than in CG context. To ascertain the methylation status of a given cytosine, methylation levels are typically converted to binary calls (methylated or unmethylated) using a binomial model, where the binomial ‘success’ parameter is fixed to the conversion rate. This rate is calculated from the unmethylated chloroplast genome. The binomial model can then estimate the probably that the observed number of methylated reads could have occurred by chance if the cytosine is actually unmethylated (van der Graaf et al., 2015). The sample size of this test corresponds to the number of reads aligned to a given cytosine position, which is directly related to the sequencing depth of the WGBS experiment. For non-CG contexts, large depths of >40x are needed to obtain high confidence methylation calls. Hidden Markov models are more advanced approaches for methylation status calling, leveraging information from neighboring cytosines without the need for information about conversion rates, and performing well in low sequencing depth regions (Taudt et al., 2016).

#### Reduced representation bisulfite sequencing

Reduced representation bisulfite sequencing (RRBS) is a more cost-efficient alternative compared to WGBS, as it only examines a representative fraction of the genome, generating DNA methylation profiles with single-nucleotide resolution (Gu et al., 2011). This technique, originally developed for studying mammals, targets CG islands for sequencing through several steps (Meissner et al., 2005). In brief, genomic DNA is treated with the enzyme MspI, which is insensitive to methylation and recognizes the CCGG sequence, resulting in the cutting of DNA into small fragments that have CG dinucleotides at the ends. The next steps are end repair, A-tailing, ligation to methylated adapters, selection and isolation of CG-rich fragments (ranging from 40 to 220 bp), followed by bisulfite conversion, PCR amplification and sequencing of the ends. Creating an RRBS library takes about nine days in total. Double-enzyme digestion allows for more accurate coverage of genome regions and more precise representation of their methylation levels (Wang et al., 2013).

Unlike mammalian genomes, plant genomes do not have clearly defined CG islands, and the traditional RRBS protocol has been modified using specific enzymes for selective amplification of specific regions of interest (Hsu et al., 2017). RRBS enables studying gene regulation in stress response, transposable element control, and crop adaptation to changing environments in any plant genome (Chen et al., 2015; Paun et al., 2019). RRBS with optimized endonuclease combinations is used to explore the impact of DNA methylation on rice responses to salt stress (Schmidt et al., 2017), and on the regulation of TEs in maize (Hsu et al., 2018). RRBS has been also applied for identifying markers that aid in the breeding and improvement of crops (Malinowska et al., 2020; Li et al., 2023). By detecting methylation patterns associated with specific agricultural traits, such as disease resistance or yield potential, valuable information for the development of new crop varieties that are more resilient to pests and diseases has been provided (Turcotte et al., 2022). RRBS revealed conserved and divergent methylation patterns that may be associated with adaptation to different environments (Mounger et al., 2021), and with the evolution of plant DNA methylation patterns (Chen et al., 2015; Paun et al., 2019).

RRBS has several main advantages over traditional BS methods (Guo et al., 2013; Gao et al., 2017). First, RRBS is more efficient, as only a small genome subset (typically 1-10%) is sequenced, targeting CG-rich regions, which reduces the need for extensive sequencing. RRBS allows for the identification of both inter- and intragenic differentially methylated regions (DMRs) with high resolution and can detect methylation changes in coding and non-coding genome regions. Because RRBS analyzes a small portion of the genome, it demands less computational power for data analysis. It also has a high level of sensitivity, requiring only 10 ng or more of non-degraded, high-quality genomic DNA. By focusing on CG sites, RRBS is capable of capturing methylation patterns in regions that are likely to be of functional importance, such as promoter regions, enhancer regions and other regulatory elements. RRBS enables the detection of tissue-specific methylation patterns through the isolation and analysis of specific cell types (Hsu et al., 2017).

Despite the listed advantages, RRBS also has limitations, such as reduced coverage of non-CG-rich regions like gene bodies or intergenic regions. It may miss small methylation differences or regions not covered by the selected regions, which results in lower resolution, compared to WGBS. RRBS may not detect methylation in repetitive elements. In addition, RRBS requires more complex library preparation and sequencing compared to traditional BS methods. Since the method relies on PCR amplification, it may introduce bias in the data, particularly in regions that are difficult to amplify. RRBS requires high-quality DNA as the reduced representation approach leads to poor sequencing of degraded DNA. Overall, RRBS is a powerful and efficient method for the analysis of DNA methylation in plants including crops. As the cost of sequencing continues to decrease, it is likely that RRBS will become an increasingly popular method for studying genome methylation patterns and their role in plant adaptation and evolution.

#### Methylation capture sequencing

Methylation Capture Sequencing (MC-seq) or Targeted BS is a capture approach that utilizes BS to obtain DNA methylation data (Morselli et al., 2021), and a cost-effective alternative to WGBS. Targeted NGS is designed to concentrate on specific genomic regions of interest (Kozarewa et al., 2015; Singh, 2022) and through its association with bisulfite treatment, can detect DNA methylation at single-nucleotide resolution (Wang et al., 2017; Morselli et al., 2021). MC-seq is similar to WGS but the sample preparation requires an extra step of target enrichment through hybridization capture with biotinylated oligonucleotide probes to capture specific regions. The method enables target enrichment specifically for methylomic regions of interest, followed by bisulfite treatment. The hybridization to specific probes can be done either before (Agilent Sure-Select Methyl-Seq, TruSeq Methyl Capture; Lee et al., 2011) or after bisulfite conversion (Roche SeqCapEpi,Wendt et al., 2018). The choice of technique depends on the number of samples, the quality and quantity of available DNA, and the biological regions of interest, as all platforms produce comparable data (Kacmarczyk et al., 2018). MC-seq gives reproducible and similar results to WGBS (Li et al., 2015a), and consists of four key steps: (1) DNA preparation (shearing, adaptor ligation), (2) hybridization capture, (3) cleaning and bisulfite conversion, and (4) NGS library preparation and sequencing (Morselli et al., 2021).

MC-seq is widely used in humans and clinical research, and has been also applied to some plant species. Heer et al. (2018) used MC-seq to detect somatic epigenetic variations in the large genome of Norway spruce (*Picea abies*), targeting over 26,000 genes. By comparing four clones grown in varying climatic conditions for 24 years, they determined the performance and reproducibility of MC-seq, and identified 334 somatic epimutations. This suggests that MC-seq has the potential to expand our understanding of methylation patterns in natural populations. Xu et al. (2019) studied maize populations, targeting DNA methylation profiling for a diverse panel of 263 maize inbred genotypes using a 15.7 Mb targeted bisulfite capture. They identified over 16 000 DMRs used for genome-wide association studies. The results showed that DNA methylation is associated with phenotypic variation of 156 traits, with some traits displaying only significant associations with DMRs but not with SNPs.

MC-seq offers several advantages over other sequencing methods, including lower DNA input requirements, cost- and time-effectiveness for large sampling or large genome size organisms, smaller datasets that demand less computational resources for storage and analysis, flexible capture size ranging from kb to Mb, scalability to handle multiple samples/sequencing runs, and the ability to capture specific regions without requiring a high-quality whole reference genome (e.g. with exon capture). In contrast to RRBS, the analyzed regions by MC-seq are not limited to the presence of the restriction site(s). Finally, bioinformatic analysis can be done with adapted WGBS pipelines or with dedicated pipelines (Vial-Pradel et al., 2019).

MC-seq approach is also limited by some points: the use of bisulfite introduces the same bias as the WGBS method; it requires careful selection of targeted regions (one possibility is to use WGBS data on a limited number of samples before MC-seq) and their bioinformatic analysis for the design of specific probes (if not publicly available that is mostly not the case for plant species), and off-target capture due to homologous genomic sequences in plant genomes (duplicated genomic regions or repeated sequences).

### Alternative methods to bisulfite conversion

Several alternatives to bisulfite treatment exist, such as the Methyl DNA Immunoprecipitation (MeDIP) approach using a 5mC antibody for methylation analysis, which can be coupled with array detection (MeDIP-chip) or sequencing (MeDIP-seq). The data generated by this method are consistent with WGBS (Wardenaar et al., 2013) and can be applied to various crops (Lafon-Placette et al., 2013; Hébrard et al., 2016; Lafon-Placette et al., 2018). However, the use of 5mC antibodies requires a significant amount of input DNA (a few micrograms), and is associated with bias toward hypermethylated regions, and cannot differentiate methylation context. Recently, a new free-bisulfite approach called Enzymatic Methyl-seq (EM-seq) has been developed (Feng et al., 2020; Hoppers et al., 2020). Bisulfite treatment causes DNA damage and degradation, resulting in libraries with high GC bias and enrichment for methylated regions. EM-seq uses enzymatic conversion of unmethylated cytosines to uracils to achieve the same sequencing product without affecting DNA integrity. The first step of EM-seq uses TET2 and an Oxidation Enhancer to protect modified cytosines from downstream deamination. TET2 enzymatically oxidizes 5mC through a cascade reaction into 5-carboxycytosine protecting 5mC from deamination. The second enzymatic step uses APOBEC, which deaminates unmethylated cytosines but does not affect 5-carboxylcytosine (5caC). The resulting sequences are similar to those generated by both WGBS and EM-seq, and can be analyzed in the same way. Libraries generated, using EM-seq, outperformed bisulfite-converted libraries in all specific measures, such as coverage, duplication, sensitivity, even GC distribution, better correlations across DNA inputs, increased numbers of CGs within genomic features, and accuracy of cytosine methylation calls (Vaisvila et al., 2021). Additionally, EM-seq is effective with lower amounts of DNA (100 pg) than WGBS. Thus, EM-seq is a promising accurate and reliable alternative to bisulfite methods, like WGBS, for detecting DNA methylation at the whole genome level (Feng et al., 2020).

### Third generation sequencing for 5mC detection

**Figure 4:**
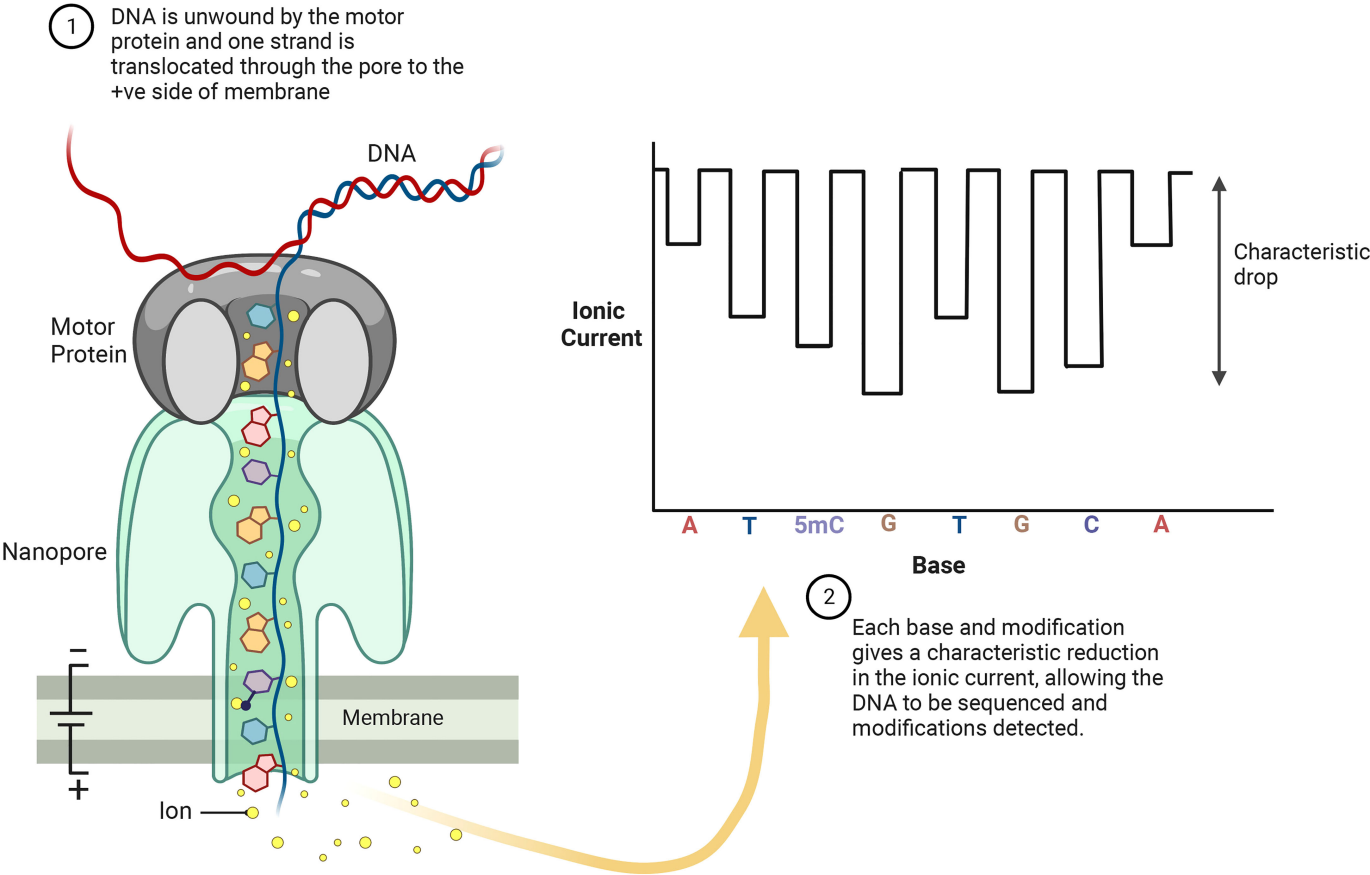
ONT sequencing platforms allow for real-time analysis of individual DNA strands as they pass through the nanopores embedded in electro-resistant membrane. Each nanopore is connected to a channel and sensor chip, which measures the electric current that flows through the nanopore. Each base and base modification produces a specific electrolytic signal allowing for detection of 5mC.

**Figure 5:**
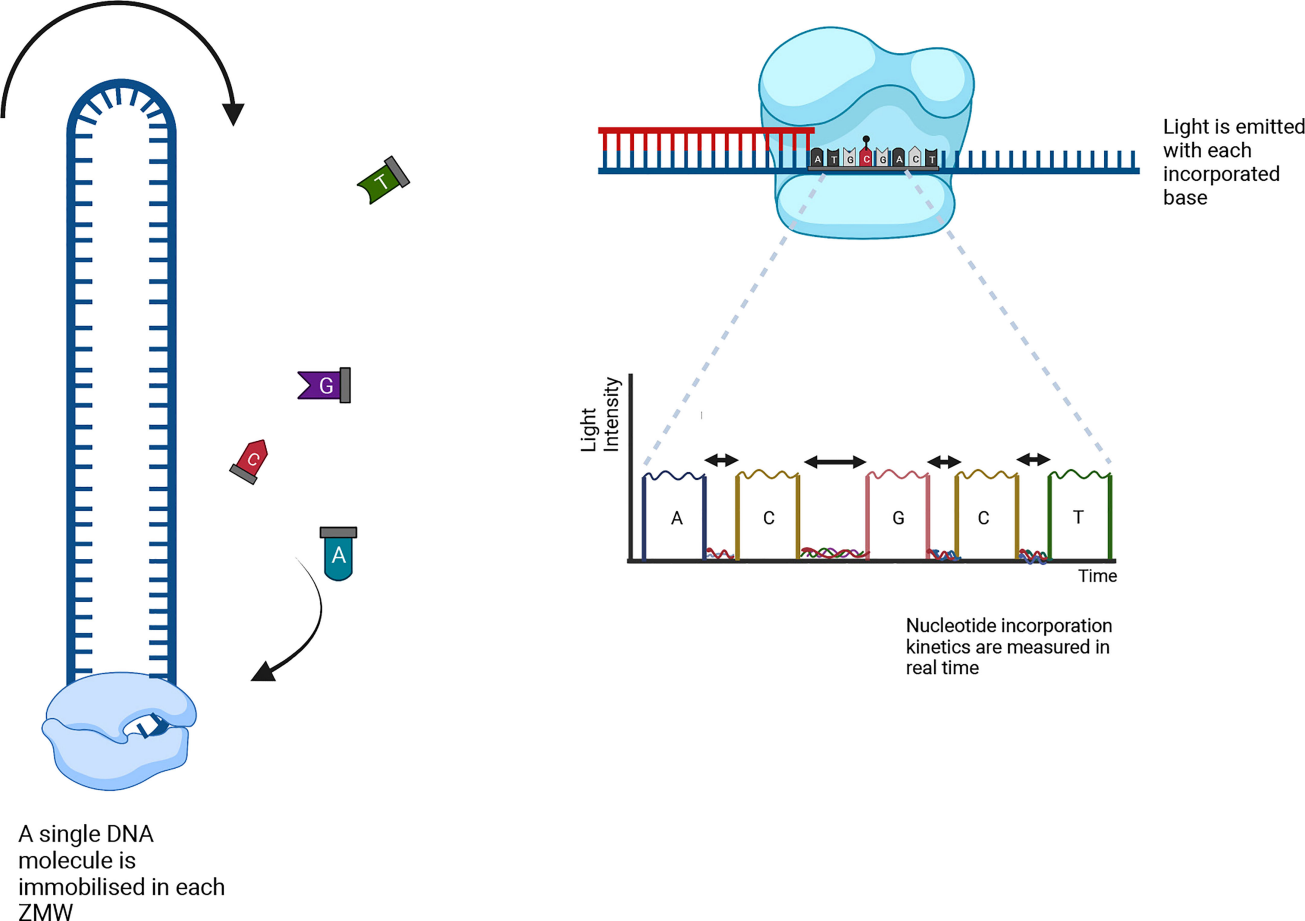
A single SMRTbell per ZMW is amplified using fluorescent-labelled nucleotides while their emission spectra are collected. The kinetic data is analyzed using bioinformatics to decipher the positions of the methylated cytosines.

There are two major long-read sequencing technologies, referred to as third generation DNA sequencing (TGS): nanopore sequencing developed by Oxford Nanopore Technologies (ONT, **Figure 4**) (Deamer et al., 2016), and single molecule real-time (SMRT) sequencing from Pacific Biosciences (PacBio, **Figure 5**). SMRT sequencing is the first TGS approach to directly observe a single molecule of DNA polymerase synthesizing a DNA strand (Levene et al., 2003; Eid et al., 2009).

PacBio sequencing detects the sequence information during the replication process of the target DNA molecule. A closed single-stranded circular DNA strand, called SMRTbell, serves as a template. The SMRTbell is loaded to a chip (SMRT cell) and diffuses into a zero-mode waveguide (ZMW), a sequencing unit that detects light signals (**Figure 5**). Four fluorescent-labelled nucleotides (red, yellow, green and blue, for G, C, T and A, respectively) are added to the SMRT cell, which generate emission spectra. The light pulse identifies the nucleotide base, and the SMRTbell replication process is recorded for all ZMWs in the SMRT cell. The recorded light pulses can be interpreted as a sequence of bases (Rhoads and Au, 2015).

The ONT core is a flow cell containing individually addressed nanopores that can be controlled in groups by an application-specific integrated circuit. Adapters are ligated to both ends of genomic DNA or RNA fragments. To ensure unidirectional single-nucleotide displacement along a DNA strand at a millisecond time scale, a processive enzyme located at the 5′-end is required. As the DNA strand passes through a pore, the shifting nucleotide sequences cause ionic changes that are detected by a sensor. These changes are segmented into discrete events that exhibit variance in mean amplitude and duration (**Figure 4**) (Jain et al., 2016). Profiling all positions of cytosine methylation contexts in plants, is a challenge for TGS. SMRT sequencing does not require base conversion to detect DNA base modifications. The kinetics of base addition is measured during sequencing, detecting over 25 base modifications, such as 6-methyladenine (6mA), 4-methylcytosine (4mC), 5-hydroxymethylcytosine (5hmC) (**Figure 5**) (Tahiliani et al., 2009; Flusberg et al., 2010). The weak effect of methylated bases over synthesis kinetics requires sophisticated statistics (Davis et al., 2013). Despite this, a good correlation to WGBS in humans has been achieved (Tse et al., 2021), moreover, the long reads allow for better mapping to the reference genome than WGBS (Miga et al., 2020).

ONT sequencing allows direct identification of DNA base modifications at single nucleotide resolution, including 5mC, 5hmC and 6mA (**Figure 4**). Liu et al. (2021) evaluated the performance of seven publicly available computational tools for methylation-calling using human Oxford Nanopore sequencing data: Nanopolish (Simpson et al., 2017), Megalodon (https://github.com/nanoporetech/megalodon), DeepSignal (Ni et al., 2019), Guppy (Oxford Nanopore Technologies: Nanopore sequencing data analysis. 2020), Tombo/Nanoraw (Stoiber et al., 2017), DeepMod (Liu et al., 2019) and METEORE (Yuen et al., 2021). METEORE is an ensemble model, which provides predictions based on two or more tools. The tools were compared using four benchmark datasets, including two human B-lymphocyte cell lines (NA19240 and NA12878), leukemia cell lines K562, and a clinical specimen of acute promyelocytic (Liu et al., 2021). For the DNA methylation ground truth, published WGBS and RRBS datasets from ENCODE are used.

Overall, the top tools are Megalodon, Nanopolish, DeepSignal and Guppy. However, these tools may still have difficulty detecting 5mCs in certain genomic regions, such as intergenic, low CG density and repetitive regions, and regions with discordant DNA methylation patterns. In terms of computational requirements, Guppy, Nanopolish and Medalodon are faster than the others, with Guppy and Nanopolish consuming the least memory. Guppy and Nanopolish predict 4% and 6% fewer CG sites than DeepSignal and Megalodon. Nanopolish can be additionally recommended as the best option due to its per-read and per-site performance criteria and the relatively low computing resource requirement. For users with available high performance computing resources, Megalodon is a good option due to its performance in more challenging areas (e.g. repetitive areas) and higher prediction of CG islands, compared to Nanopolish and Guppy. A comprehensive comparison of these tools is available at https://nanome.jax.org/.

Detecting DNA methylation using PacBio sequencing data presents limited bioinformatic options, but recent developments have expanded the field. PacBio SMRT Link (v11.0) software now includes a machine learning approach for 5mC detection in CG contexts (Wenger et al., 2019), while Tse et al. (2021) developed holistic kinetic model (HK model), a convolutional neural network that correlates highly with BS results. More recently, Ni et al. (2022) published ccsmeth, a deep learning method that detects 5mCs in CG contexts from PacBio circular consensus sequence (CCS) subreads with greater accuracy, than the HK model on amplified and M.SssI-treated DNA. The ccsmeth method uses a recurrent neural network with bidirectional Gated Recurrent Units (GRUs) with attention.

Third generation SMRT technology has successfully mapped N^4^mC and N^6^-adenine (6mA) in the crop plants *Ficus carica* (fig) (Usai et al., 2020) and *Casuarina equisetifolia* (Coastal She-oak) (Ye et al., 2019). Meanwhile, genome-wide mapping of 6mA has been achieved for Arabidopsis at different developmental stages and rice with over 100-fold coverage enabling detection (Liang et al., 2018; Zhang et al., 2018b). Although base modifications in Coastal She-oak and fig were identified at 14-fold and 74-fold coverage, respectively, using the PacBio and KineticsTools (Ye et al., 2019; Usai et al., 2020), no reports of 5mC detection using SMRT technology in plant genomes have been found to date. Recent advancements in ONT technology led to the development of Deepsignal-plant, a tool that enables detection of DNA methylation in all three contexts for plant genomes. Evaluations of Arabidopsis and rice genomes showed high correlations between Deepsignal-plant and WGBS for CG (p>0.98) and CHG (p>0.93) contexts, but relatively lower correlations for CHH (p>0.82). Interestingly, the use of ONT revealed 1-5% more methylated cytosines compared to short-read based WGBS, with most of them located in centromeres, pericentromeric, and telomeres (Ni et al., 2021).

### Immuno-based techniques to monitor DNA methylation nuclear patterns and global levels: Immunofluorescence and ELISA-like assays

Analysis of nuclear distribution patterns of 5-methyl-deoxy-cytidine (5mdC) provides a powerful approach for investigating global DNA methylation dynamics in specific cell types of a particular tissue/organ during development or under different environments. Unlike other assays that only quantify the percentage of methylated cytosines, this method allows for distinguishing methylation patterns among different cell types within the same organ.

Access to a specific and robust 5mC antibody (commercially available) permits its successful application in immunofluorescence (IF) assays to study individual cells/tissue sections in various plant species (Meijón et al., 2009; Solís et al., 2012; Conde et al., 2013; Rodríguez-Sanz et al., 2014b; Solís et al., 2014; Solís et al., 2015; Rodríguez et al., 2016; Conde et al., 2017; Gomez-Cabellos et al., 2022; Silva et al., 2022), and by whole-mount approaches in certain organs (She et al., 2018). Confocal laser scanning microscope (CLSM) analyses of immunolocalized 5mdC reveal distinctive DNA methylation distribution patterns in the nucleus linked to cell differentiation, proliferation, or reprogramming events in specific developmental programs (Testillano and Risueño, 2016). Versatility and feasibility of this approach have been demonstrated for different plant species and cell types, regardless of characteristics like hardness, heterogeneity, cell accessibility and tissue compactness for *in situ* cellular analysis in sections. Quantification of 5mdC IF intensity through appropriate image software and techniques permits the assessment of changes in global DNA methylation levels among different cell types or experimental conditions (Testillano et al., 2013). This method has been applied to investigate DNA methylation dynamics during plant reproductive organ development, as well as *in vivo* and *in vitro* embryogenesis (Ribeiro et al., 2009; Solís et al., 2012; El-Tantawy et al., 2014), in programmed cell death during cork oak differentiation (Inácio et al., 2018), and in tapetal cells of rapeseed anthers (Solís et al., 2014).

**Figure 6:**
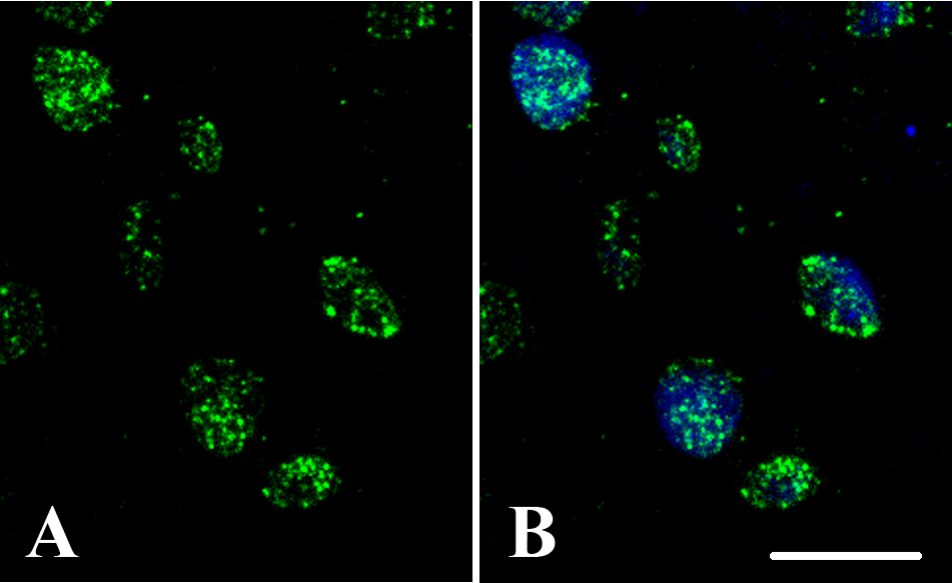
DNA methylation nuclear distribution patterns. *Quercus suber* proliferating embryogenic masses. Confocal images of: **(A)** 5mdC immunofluorescence (green), and **(B)** merged image of DAPI (blue) and 5mdC immunofluorescence (green). Bar: 10 µm.

In plant samples, the 5mdC IF signal, typically detected in sections of plant organs and tissues, are often very thick, making whole-mount approaches difficult and species-dependent. Sample processing before IF is a critical step in the success of this technique. Fixation in paraformaldehyde followed by dehydration in acetone or methanol and embedding in acrylic resins that polymerize at low temperature, like Technovit^®^ 8100, is a very convenient method that provides good structural preservation, even at the subcellular level, and adequate antigenic reactivity to 5mdC antibodies. Sections of 1-2 µm thickness can be obtained from resin-embedded samples, which permits getting IF microscopic images with high resolution, even in conventional epifluorescence microscopes, and subnuclear level analysis of 5mdC distribution patterns. Thicker sections can also be obtained through paraffin embedding or without embedding by vibratome or cryostat sectioning. Vibratome permits sectioning fresh and fixed samples directly but requires highly homogeneous and soft tissues, limiting its application only to some plant samples. Nevertheless, harder samples, such as lignified stems, have been successfully sectioned (Inácio et al., 2018). Cryostat sectioning requires fixation, cryoprotection and freezing of samples, as sectioning is performed usually at temperatures between -20 and -40 °C. Although both techniques are much simpler than resin embedding and sectioning, these sections are thicker (around 30-40 µm in vibratome and 10-20 µm in cryostat and paraffin embedding, depending on the type of sample), and several strong permeabilization steps are necessary prior to IF to aid antibody penetration and binding to the target, such as freezing-thawing, dehydration-rehydration, and mild cell wall enzymatic digestion. It is important to note that thick sections do not provide sufficient IF image resolution for visualizing distribution patterns at the subnuclear level but can be useful for histological studies. Observation with CLSM allows to obtain optical sections and avoid the out-of-focus fluorescence in the thick vibratome and cryostat sections. Semithin resin sections (1–2 μm thickness) can be also analyzed by both CLSM and epifluorescence microscopes. The use of CLSM for the analysis of 5mdC IF assays greatly improves signal intensity and resolution, providing high quality images of the nuclear distribution of DNA methylation patterns (**Figure 6**). The CLSM usually contains a wide set of image analysis tools for further examination of IF signals. After processing and sectioning, 5mdC IF assays involve standard incubation steps with a primary antibody followed by the application of fluorochrome-conjugated secondary antibody. A critical step of 5mdC detection is pretreating the sections with HCl, which partially denatures DNA and facilitates optimal exposure of target epitopes to the antibodies. Frequently, a final step to stain DNA by a fluorescent dye, DAPI or Hoestch, is used, allowing for easier localization of nuclei through excitation at a different wavelength (UV) than that used for 5mdC detection. This step enables merging of individual fluorescent signals of different colors into the same image for an accurate analysis of DNA methylation patterns, their subnuclear distribution, and link with chromatin condensation. To ensure the accuracy of the information obtained from the IF experiments, technical controls are necessary. Most common controls for 5mdC IF experiments are avoidance of the HCl denaturation step or primary antibody. Immunodepletion assays can also be performed, in which the antibody is pre-blocked with the antigen (5mdC) *in vitro* and then used for IF experiments. Due to the autofluorescence of certain components of the cell wall, a negative control without the secondary antibody can be used.

CLSM with fixed parameters, such as laser excitation and sample emission capture settings, during image acquisition in all IF preparations, enables accurate comparison of IF signals from various cells or conditions. This is achieved by quantifying fluorescence intensities to estimate changes in global DNA methylation levels across cells, developmental stages, or conditions (Testillano et al., 2013). To make reliable and accurate comparison, confocal optical sections of each IF preparation are collected at the same z-intervals and the same total number of optical sections. By adhering to these conditions, maximum projection images can be generated and used for fluorescence intensity quantification. Various image analysis software, either from CLSMs or other free and commercial tools (Image J/Fiji and Photoshop), can be used for this purpose.

The 5mdC antibodies also enable the analysis of global levels of DNA methylation among various plant samples or conditions by ELISA-like immunoassays. Although most commercially available kits are designed for animal DNA samples, they can still be used to compare DNA methylation in plant samples. Even though the 5mdC nucleotide is not different between plants and animals, the genomic DNA methylation context is very different between those two groups with plants having methylation in CHG and CHH contexts in addition to CG contexts. Nevertheless, commercial kits allow to compare DNA methylation in multiple samples, providing an accurate quantification of 5mdC in several DNA samples simultaneously and in a short period of time. Briefly, DNA is bound to strip wells with a high DNA affinity, followed by the capture of the DNA methylated fraction using 5mdC antibodies further recognized by an enzyme-conjugated secondary antibody (an “indirect” ELISA). A fluorometric or colorimetric substrate is then added to yield a measurable signal proportional to the 5mdC amount. The methylated fraction of DNA is estimated by quantifying the optical density/fluorescence intensity with a microplate spectrophotometer at the appropriate excitation and emission wavelengths for the assay type (colorimetric or fluorometric). After normalization with positive and negative controls (DNA samples containing zero or a known percentage of 5mdC), DNA methylation can be compared among samples and conditions. Absolute quantification of 5mdC can be inferred by generating a standard curve with the controls. The global DNA methylation can be extrapolated by multiplying the percentage of methylated cytosines by the total cytosines per genome length of the plant species of interest. In the case of absolute quantifications, commercial kits control only bear CG methylation and exclude other sequence contexts. It is advisable to use control with cytosine content as close as possible to the analyzed samples to minimize the magnitude of the mathematical corrections, and appropriate biological and technical replicates to get statistically robust data. This technique has been applied to analyze global DNA methylation levels in various plant conditions and species, from herbaceous to woody plants.

Global DNA methylation changes are observed during development of barley and rapeseed microspore embryogenesis cultures, with significant decreases found after treatments with the demethylating agent 5-azacytidine (Solís et al., 2015). In maize, global DNA methylation alterations are linked to metabolic changes induced by microbial-based biostimulants (Lephatsi et al., 2022). Differences in global DNA methylation are also seen throughout the infection process in pseudo-organs developed by root-knot nematode infection in Arabidopsis and tomato (Silva et al., 2022). In *Quercus suber*, cork samples show unequal global DNA methylation contents according to different industrial qualities (Ramos et al., 2013). During somatic embryogenesis in *Q. suber*, there is a global DNA methylation decrease accompanying nuclear remodeling in early embryo cells (Rodríguez-Sanz et al., 2014a).

### Determination of 5mC and other non-canonical nucleosides with mass spectrometry

**Figure 7:**
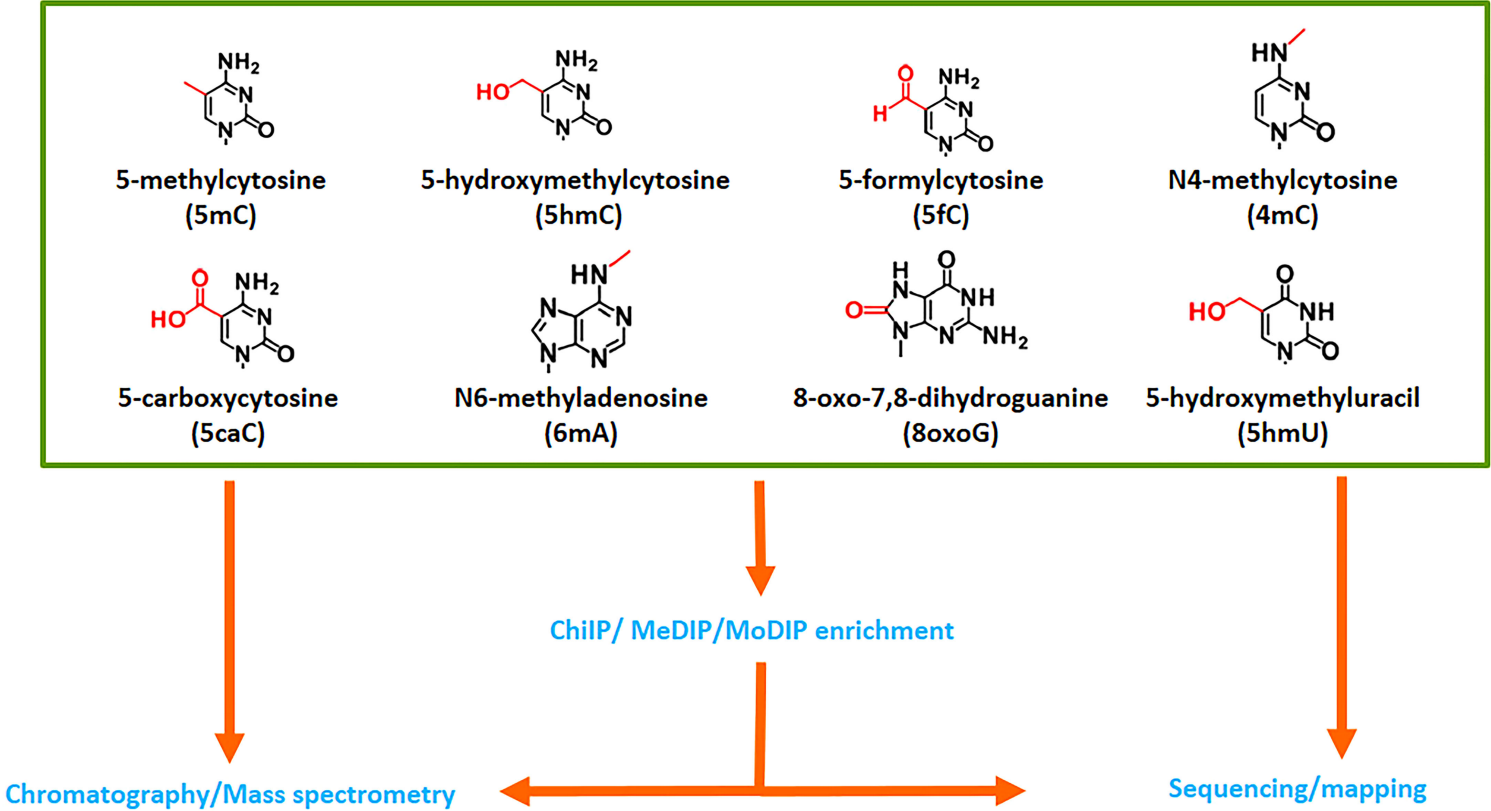
Variants of DNA nucleobases in plants. 5-Methylcytosine is the most well-known modified nucleobase, but seven others have been documented in plants. Mass spectrometry is a powerful tool for their detection, while some sequencing techniques can also detect and map their localisation in the genome. However, this approach is restricted to species with a reference genome.

All four bases of DNA can be subject to multiple changes. To date, over 52 non-canonical nucleosides have been identified in different organisms (Sood et al., 2019; https://dnamod.hoffmanlab.org), including plants (**Figure 7**). The most common epigenetic modification is the methylation of cytosines and adenosines. In eukaryotic genomes, 5mC is widely present and the best studied modification. In mammals, 5mC is converted to 5hmC, and further to 5-formylcytosine (5fC) and 5caC by TET enzymes, which have different expression patterns and targets during development. TETs can also oxidize thymine producing 5-hydroxymethyluracil (5hmU), which can also be formed through enzymatic or spontaneous hydrolytic deamination of 5hmC (Ito et al., 2011; Pfaffeneder et al., 2011; Fu and He, 2012). Although TET-like enzymes have been reported in plants, their role in epigenetic modifications remains experimentally unverified (Mahmood and Dunwell, 2019). Contradicting reports exist on the presence or absence of 5mC derivatives in plant genomes. Early studies are based on indirect or semi-quantitative measurements. A dot-blot assay estimated low but measurable amounts of 5hmC (∼0.068–0.075% of total cytosine nucleotides in the genome) in Arabidopsis leaves and flowers (Yao et al., 2012). The presence of 5hmC has been established in Arabidopsis, rice and *Glycine max* through measurement of [3H] glucose transfer to 5hmC by recombinant β-glucosyltransferase (Terragni et al., 2012). The existence of 5hmC is also confirmed in *Cucumis sativus* and *Brassica oleracea* using an antibody-based colorimetric ELISA-like reaction (Moricová et al., 2013) and in three rice cultivars through a dot-blot assay or liquid chromatography-multistage mass spectrometry (LC-MS^3^) (Wang et al., 2015b). Other studies reported the absence of 5mC oxidation products in Arabidopsis (Jang et al., 2014) or levels below 0.01% (Erdmann et al., 2015). A more sensitive and reliable approach using HPLC fraction enrichment and stable-isotope dilution LC-MS^3^, detected all 5mC oxidation products (5hmC, 5fC and 5caC), and 5hmU in Arabidopsis genomic DNA (Liu et al., 2013). Tang et al. (2014b) employing Girard’s reagents derivatization-based LC/ESI-MS/MS method, identified and quantified 5fC and 5caC in level, ranging from 2.1-4.7 per 106 dG and 0.2-3.4 per 106 dG, respectively, in genomic DNA from *Arabidopsis*, tomato, maize and rice. 5hmC, 5fC, 5hmU and 5caC (except 5caC) are also detected by IF and quantified using a two-dimensional UPLC-MS in the genome of Norway spruce (Yakovlev et al., 2019). Alternatively, 5hmC can be spontaneously produced by oxidative damages resulting from reactive oxygen species (ROS). Therefore, a trace amount of 5hmC can be present in plant genomes without corresponding enzyme activities.

The study by Moricová et al. (2013) found no significant impact of ascorbic acid on DNA hydroxymethylation in *C. sativus* cultures and a decreasing trend in *B. oleracea* protoplast cultures, without any ROS signal being detected. None of the oxidized derivatives of 5mC correlated with the levels of the products of DNA damage (uracil and 8-oxo7,8-dihydroguanine, 8oxoG) in spruce samples (Yakovlev et al., 2019), indicating that the changes in DNA hydroxymethylation are mainly independent of DNA damage and probably due to enzymatic modifications with a small contribution from oxidation or spontaneous deamination. To accurately quantify 5hmC, 5fC, 5hmU and 5caC, it is strongly recommended to simultaneously monitor the levels of non-enzymatic DNA oxidation (8oxoGe) and deamination (uracil).

Ultra-high-performance LC coupled with tandem MS is the preferred method for detecting and measuring DNA modifications due to its high sensitivity and specificity. It is considered the “gold standard” (Raiber et al., 2017) for quantitatively detecting modified DNA bases. Various DNA extraction methods are suitable for subsequent MS analysis, but strong buffers and excess of mono- and bivalent metal cations should be avoided in the sample. The analyses can be performed using a modified version of a previously described method (Starczak et al., 2022) (**Supplementary data**). In the future, nanopore sequencing could be used to identify the location of different epigenetic marks in plant genome (White and Hesselberth, 2022), but it will only be applicable to plant species with a reference genome.

### Avoiding DNA contamination in epigenetic studies

DNA contamination (DC) can be a challenge in epigenetic research, including MS, MSAPs and sequencing-based methods. DC has been reported for many reference genomes (Stocks et al., 2019; Lupo et al., 2021; Cornet and Baurain, 2022) due to various factors like biological causes (plant microbiome, chimeric organisms, and taxonomic errors), experimental issues (DNA isolation and sequencing contamination), and computational issues (*in silico* processing, metagenomic binning/assembly and chimeric sequences). Several strategies have been employed to address DC in epigenetic research. Utilizing sterile *in vitro* cultures can reduce DC, but complete elimination of endophytic microorganisms in plants seems to be unfeasible as microorganisms present even in supposed “axenic” cultures (Esposito-Polesi et al., 2017). Bacterial DNA can be removed by digesting it with the restriction enzyme DpnI, followed by size-exclusion ultrafiltration (Xiong et al., 2019). This approach is based on the high number of methylated adenines in bacterial DNA compared to other organisms, and methylation-sensitivity of DpnI that cuts DNA at the GATC sequence only when the adenine is methylated (Lacks and Greenberg, 1975). Microfiltering to separate plant and microbiome cells prior to DNA isolation has also been used, leading to a reduction of bacterial DNA, though not a complete elimination (Aliche et al., 2021). Currently, no similar strategies have been reported for selectively removing DNA from other microbiome components, like fungi, archaea, and protists.

The main caveat of MS is its inability to discriminate the source in a given plant DNA sample, which can pose challenges when modifications are of very low abundance. Strategies to reduce DC issues include avoiding bacterial enzymes during the sample preparation process, incorporating blank/mock samples in each experiment, utilizing axenic plants, and implementing protocols for bacterial cell or DNA elimination. However, unlike sequencing data, there are no *post-hoc* options for removing DC from MS data. DNA contamination is also a concern in sequencing-based methods, and various programs like ConFindR, CheckM, EukCC and BUSCO can detect it, though these programs have some limitations (Cornet and Baurain, 2022). While DC should be taken seriously in epigenetic studies, it is worth noting that epigenetic changes in the microbiome can also have important implications for plant development and stress resistance (Vannier et al., 2015).

All plants are holobionts, or a community of the plant with its endogenous and exogenous microorganisms (microbiome). The term hologenome has been proposed to explain the complex genetic regulation in holobionts (Rosenberg and Zilber-Rosenberg, 2018). Epigenetic changes in any part of the hologenome can affect plants. Therefore, keeping plant and microbiome DNA together in MS studies is a more accurate reflection of the natural conditions of all plants.

### Methods for modulating DNA methylation in model and crop species

#### Generation of DNA methylation mutants using genetic and epigenetic means

Understanding the molecular basis of DNA methylation changes in crops requires direct interference with DNA methylation marks and the associated pathways involved in its addition, maintenance, and removal. Possible methods for influencing DNA methylation levels in crops include genetic, epigenetic, and pharmacological approaches.

Loss-of-function genetic mutants of trans-acting methylation factors are essential for DNA methylation studies (e.g. Stroud et al., 2013), and public collections for model species like Arabidopsis (Calhoun et al., 2019) or *Medicago truncatula* (Tadege et al., 2008) are available. However, such collections are rare for crops, except for several species like rice (Kurata and Yamazaki, 2006; Wang et al., 2013), tomato (Saito et al., 2011) or maize (Andorf et al., 2016; Lu et al., 2018). Mutants in epigenetic regulatory genes can also be obtained from gene mapping projects as exemplified by the histone demethylase SIX-ROWED SPIKE3 (VRS3) (Bull et al., 2017; van Esse et al., 2017). Tilling populations are another valuable source of mutants for many crops, including main cereals. They offer a straightforward means to isolate a range of mutant alleles of various strengths (Kurowska et al., 2011; Tadele, 2016).

Recently, genetic mutations are induced by nucleases targeted to specific endogenous DNA sequences, such as artificial Zinc fingers (ZF), Transcription activator-like effector nucleases (TALENs), or the Clustered Regularly Interspaced Short Palindromic Repeats (CRISPR)/Cas9 (Podevin et al., 2013; Chen et al., 2019). CRISPR/Cas9 has inborn nucleolytic activity, while other systems require fusion with a nuclease. However, CRISPR-based mutagenesis uses only simple cloning, short hands-on time and low costs, making it a popular and rapidly developing method (Chen et al., 2019). However, practical application of these new genomic techniques in improving crops is subject to strict regulation in some countries.

Besides mutagenesis, epigenome editing can be achieved through a fusion of modified ZFs, TALEs and CRISPRs with enzymes/domains. ZF fusions with components of the RdDM pathway induce DNA methylation and silencing of the floral repressor *FLOWERING WAGENINGEN* (*FWA*) in Arabidopsis (Johnson et al., 2014; Gallego-Bartolomé et al., 2019). Besides, combining non-nucleolytically dead Cas9 variants (dCas9) with SunTag (Tanenbaum et al., 2014) and enzyme domains allows several effectors to act on a single target locus, including targeting of DNA methylation into the FWA promoter using either the dCas9 fusion with *Nicotiana tabacum* DRM (Papikian et al., 2019) or CG-specific bacterial methyltransferase SssI (Ghoshal et al., 2021). Importantly, the Cas9 SunTag fusion with SssI yielded greater heritability and lower off-target methylation than ZF-based SssI silencing (Liu et al., 2021). The system can also induce DNA demethylation using the human TET1 catalytic domain fused to dCas9 or ZF (Gallego-Bartolomé et al., 2018). Recently, epigenome editing of an *S* gene enhanced cassava resistance to *Xanthomonas axonopodis* pv. *manihotis* (Veley et al., 2021). Although a limited number of studies have applied targeted DNA (de)methylation changes in plants, this seems a very promising direction. Further research is needed to refine key parameters such as reducing off-target events or increasing the heritability and predictability of DNA changes in specific genome regions.

Silencing constructs like RNA interference (RNAi) (Přibylová et al., 2019; Zicola et al., 2019), artificial microRNAs (amiRNA), or virus-induced gene silencing (VIGS) (Bond and Baulcombe, 2015; Atsumi et al., 2021) can weaken or temporary inactivate DNA methylation factors. Such mutants, developed for multiple crops, offer numerous advantages, including variable silencing strengths and simultaneous targeting of multiple homologs.

#### Chemically-induced methylation changes as the fastest method in the non-model species

Pharmacological approaches can be a viable alternative when other methods are difficult or not applicable. Epigenetic drugs typically induce weaker changes than genetically-induced depletion of DNA methylation, occur more randomly in the genome, and are rapidly restored in most tissues (Baubec et al., 2014; Griffin et al., 2016; Nowicka et al., 2020). A variety of chemicals can inhibit chromatin modifiers in plants (Zhang et al., 2013; Pečinka and Liu, 2014), resulting in changed chromatin patterns. Some chemicals and their use have already been described in detail; therefore, we focus on some novel aspects here. Classically, non-methylable cytidine analogs from the 5-azacytidine family, including 5-azacytidine, 2’-deoxy-5-azacytidine, 5-azacytidine, 2’-deoxy-5-azacytidine and zebularine, have been used to reduce DNA methylation in many plant species. Chemical stability and degree of demethylation vary among these analogs (Liu et al., 2015; Nowicka et al., 2020). The effects tend to be stronger in actively dividing tissues and are transient due to the *de novo* demethylation activity of RdDM (Baubec et al., 2014), which may be linked to the lower chemical stability of 5-azacytidine-type drugs. Other more stable 5-azacytidine derivatives (5,6-dihydro-5-azacytidine, 2’-deoxy-5,6-dihydro-5-azacytidine, α-2’-deoxy-5,6-dihydro-5-azacytidine) have been tested in plants.

While a reduction of DNA methylation is seen in some tree cultures (Baránek et al., 2019), no obvious effect in transcriptional activation of transcriptionally silent reporter locus is found in *Arabidopsis* (Nowicka et al., 2020). Recent study revealed that 5-azacytidine drugs are actively transported into plant cells by EQUILIBRATIVE NUCLEOSIDE TRANSPORTER 3 (ENT3) and covalently trap MET1 to the DNA molecule (Prochazkova et al., 2022), creating a DNA-protein crosslink that triggers a DNA damage signal and requires repair. Hence, DNA methylation inhibitors of the 5-azacytidine family reduce DNA methylation at least transiently, as their effect is more complex and leads to DNA damage.

Other substances alter DNA methylation without directly interacting with DNA methyltransferase. The most common strategy is to disrupt the production of the S-adenosyl-1-methionine (SAM), a methyl group donor transferred by DNA methyltransferases (Roje, 2006). Dihydroxypropyladenine (DHPA) inhibits the regeneration of SAM precursors, such as homocysteine and adenosine, by inhibiting the enzyme SAHH1. DHPA is involved in DNA demethylation (Kovařík et al., 1994), reversion of transgene epigenetic silencing (Baubec et al., 2010) and deregulation of flowering genes coupled with altered flower morphology in tobacco (Fulneček et al., 2011). Sulfamethazine is less used substance that affects SAM levels by disrupting methyl sources depending on folate biosynthesis pathways, and suppresses transgene epigenetic silencing in *Arabidopsis* (Zhang et al., 2012). The natural product sinefungin competes with SAM for its binding site (Selberg et al., 2019). Development of epigenetically active substances for plant studies is advancing with research in human medicine, particularly in cancer treatments. Ganesan et al. (2019) summarize all the recent advances in this area and mention new perspective chemicals.

DNA methylation can also be affected by histone acetylase/deacetylase inhibitors. This is possibly due to the crosstalk between DNA methylation and histone modifications that can mutually reinforce each other actions and act coordinately in gene silencing (Dobosy and Selker, 2001; Irvine et al., 2002). For example, treatment with trichostatin A, a class I and II histone deacetylase inhibitor, increased histone H4 acetylation, while decreasing both global H3K9me2 and DNA methylation during mitosis in maize root tip cells (Yang et al., 2010). Similarly, treatment with the G9a histone methyltransferase inhibitor BIX-01294 (homologous of the plant SUVR4-HKMTs and responsible of H3K9 methylation) reduced global H3K9me2 and DNA methylation in microspore embryogenic cultures of rapeseed and barley (Berenguer et al., 2017). Chen et al. (2022) also reported reduced DNA methylation using trichostatin A, but the effect may be locus-specific since no activation was observed from the transcriptionally silenced multicopy reporter gene (TSGUS) in *Arabidopsis* (Nowicka et al., 2020). Sirtuins, a class III histone deacetylases, are involved in plant stress responses and their functional properties with an emphasis on epigenetics were thoroughly reviewed by Kosciuk et al. (2019). Specific substances can modulate the activity of sirtuins, and their application has also caused DNA methylation changes in treated subjects (Baránek et al., 2021).

### Success of the methods between model and crop plants

Given the numerous techniques available for DNA methylation profiling, it is crucial to use a systematic approach when designing experimental conditions to assess epigenetic variations (Paun et al., 2019). Although initial insights into methylation patterns come from the *Arabidopsis* methylome, much more remains to be discovered, considering the wide variation in genome methylation across flowering plant species (Niederhuth et al., 2016). Choosing the right technique depends on factors such as genome size and complexity and the availability of reference genomes. This section provides a brief overview of the use of different techniques in both model and crop species.

One of the most common approaches used for analyzing DNA methylation in plants, MSAP, was first applied in rice (Xiong et al., 1999). Versatility of this method, regardless of the genome size or the presence of a reference genome (Chwialkowska et al., 2017), makes it widely applicable in both model and non-model plants. In *Arabidopsis*, MSAP has been used to detect stress-induced methylation changes, such as cryopreservation (Wang and He, 2009), cadmium stress (Li et al., 2015b) and sulfur dioxide stress (Yi and Li, 2013), also in crops, such as faba bean under dehydration (Abid et al., 2017), *Sesamum indicum* under drought and waterlogging (Komivi et al., 2018), and maize under sulfur and chlorine stress (Zenda et al., 2018). Additionally, differences in cytosine methylation between *Pinus pinea* individuals and populations, and *Laguncularia racemosa* populations have been studied (Lira-Medeiros et al., 2010; Saez-Laguna et al., 2014).

The availability and affordability of sequencing techniques lead to the replacement of polyacrylamide gels with high-throughput sequencing using NGS and automated MSAP-Seq data analysis. This approach is effective in crops with large, complex and highly repetitive genomes. It has been used to study barley drought tolerance (Chwialkowska et al., 2016), and the methylation of two somatic wheat mutants (Baránek et al., 2016). Guarino et al. (2020) demonstrated the effectiveness of MSAP-NGS in assessing epigenetic variation in species with unavailable genome sequences, as demonstrated by their study of stress-related epigenetic changes in white poplar.

RRBS, originally designed for mammalian methylome analysis with MspI enzyme, required protocol adjustment for plant methylome study, since plant genomes lack typical CG islands (Andrews, 2013). Chen et al. (2015) utilized SacI/MseI double-digested fragments for modified RRBS analysis of *Brassica rapa*, which randomly covers ~2% of cytosines. Hsu et al. (2017) developed an *in silico* pipeline for selecting specific enzymes and used MseI- and CviQI-digested fragments to analyze tissue-specific mCHH islands in maize. Schmidt et al. (2017) improved the analysis of plant methylomes using Plant-RRBS, which employs optimized double restriction endonuclease combinations (MspI-DpnII or MspI-ApeKI) for plant methylome analysis, including rice. This approach offers advantages over using a single restriction endonuclease that only covers a limited number of cytosine positions, hampering subsequent comparative analyses, as seen in the study of *Quercus lobata* Née (Gugger et al., 2016). Furthermore, Malinowska et al. (2020) introduced WellMeth, a RRBS pipeline specifically designed for methylation analysis of plants with available high-quality reference genomes. WellMeth enables quantification of methylation at single-base resolution, identification of DMRs and cites, and has been successfully applied to spring barley.

Compared to RRBS, WGBS is a more expensive technique, particularly for large genomes, and it is restricted to species with high-quality reference genomes. Nonetheless, WGBS has been successfully used for studying methylation patterns in *Arabidopsis* (Cokus et al., 2008; Lister et al., 2008) but also for different crop methylomes including *B. rapa* (Liu et al., 2018; Takahashi et al., 2018), maize (Gent et al., 2013; Regulski et al., 2013), *Morus alba* (Li et al., 2020), *Bruguiera gymnorhiza* (Miryeganeh et al., 2022), *Populus trichocarpa* (Liang et al., 2014), *Populus euphratica* (Su et al., 2018). Combining low-coverage WGBS with high-coverage targeted capture of specific loci after bisulfite conversion provide a cost-effective approach for analysing specific mutant alleles and their impact on the maize methylome (Li et al., 2014). This approach can be useful for similar analysis in other crops with large, repetitive genomes, and is less expensive than regular WGBS.

The immunolocalization approach, a visual and qualitative technique, can be applied to all plant species. It has been used to detect DNA methylation patterns in *Arabidopsis* (Fransz et al., 2002; Tessadori et al., 2007), 5mC distribution patterns in *Secale cereale* and *Capsella bursa-pastoris* (Kalinka and Achrem, 2020; Gomez-Cabellos et al., 2022), and changes in histone methylation during microspore reprogramming to embryogenesis in rapeseed and barley (El-Tantawy et al., 2014; Rodríguez-Sanz et al., 2014a), and during stress-induced pollen reprogramming in rapeseed (Solís et al., 2012). Santamaría et al. (2009) analyzed genomic DNA methylation patterns during bud set and bud burst in *Castanea sativa* and Pérez et al. (2015) during different embryo developmental stages in cork oak.

## Conclusion and future prospects

Over the last two decades, the field of plant epigenetics has become increasingly important in understanding the genetic and molecular mechanisms underlying key agronomic traits, such as growth and development, disease resistance, and adaptation to environmental stress. This has led to development of various techniques for studying DNA methylation, aiming at a balance between the need for high-resolution data, cost-effectiveness and technical complexity. Each method has its own advantages and disadvantages, and the selection of the appropriate techniques depends on the research question and resources available. There is a growing need for affordable and accessible DNA methylation analysis kits, less technically complex, that would allow a wide range of researchers to conduct epigenetic studies on important agronomic traits. Furthermore, NGS has become the standard technology for DNA methylation analysis, due to its affordability, however, the development of user-friendly bioinformatic tools to analyze epigenetic variations and their effects on plants is still a very critical point. These advancements can deepen our understanding of the role of epigenetics in plant biology and encourage breeders to explore all sources of variation impacting plant phenotype.

## Author contributions

All authors listed have made a substantial, direct, and intellectual contribution to the work, and approved it for publication.
