# Supplementary material for: Exploring the crop epigenome: a comparison of DNA methylation profiling techniques
Source: Front Plant Sci. 2023 May 30;14:1181039. doi: 10.3389/fpls.2023.1181039 (PMC10306282; doi:10.3389/fpls.2023.1181039)
Supplement: Supplementary file 1 [file DataSheet_1.pdf]

Supplementary Material

Exploring the crop epigenome: a comparison of DNA methylation profiling techniques

Dolores Rita Agius[1,2], Aliki Kapazoglou[3], Evangelia Avramidou[4], Miroslav Baranek[5], Elena Carneros[6], Elena Caro[7], Stefano Castiglione[8], Angela Cicatelli[8], Aleksandra Radanovic[9], Jean-Paul Ebejer[1], Daniel Gackowski[10], Francesco Guarino[8], Andrea Gulyás[11], Hans Hoenicka[12], Vera Inácio[13], Frank Johannes[14], Erna Karalija[15], Michal Lieberman-Lazarovich[16], Federico Martinelli[17], Stéphane Maury[18], Velimir Mladenov[19], Leonor Morais-Cecílio[20], Norbert Hidvégi[11], Aleš Pečinka[21], Eleni Tani[22], Pilar S. Testillano[6], Dimitar Todorov[23], Luis Valledor[24], Valya Vassileva[23]*

*** Correspondence:** Valya Vassileva: valyavassileva@bio21.bas.bg

SUPPLEMENTARY TABLE 1. Summary of the main steps of the experimental pipelines and approaches used for NGS data analysis.

Guarino et al. (2020)	Chwialkowska et al. (2017)	Baránek et al. (2016)
MSAP analysis up to pre-selective PCR	MSAP analysis up to selective PCR	MSAP analysis up to pre-selective PCR
Library preparation for NGS analysis of amplicons and deep amplicon sequencing	Library preparation for NGS analysis of amplicons and deep amplicon sequencing	Library preparation for NGS analysis of amplicons and deep amplicon sequencing
Read clean up trimming and excluding low quality sequences	Read clean up trimming and excluding low quality sequences	Read clean up trimming and excluding low quality sequences

<table>
<tr><td colspan="2">De novo genome assembly including sequence obtained from all samples and from both MspI and HpaII digestions and mapping of reads (of each sample and of each digestion) on reference genome assembled</td><td>Read filtration for presence of HpaII adapter and clipping of adapters, mapping to the reference genome of reads containing CGG tags on the ends</td><td>Assembly of sequences and mapping of reads on reference genome</td></tr>
<tr><td colspan="2">Comparison of number of contigs obtained by each sample and for each digestion (Venn analysis)</td><td>Functional annotation of reads</td><td>Comparison among different samples and catching of contigs with identical or differing scenario</td></tr>
<tr><td colspan="2">BLAST analyses of contigs of each sample and for digestions using as reference genome that of Arabidopsis thaliana</td><td>Normalization of counts for each CCGG site</td><td></td></tr>
<tr><td>Comparison of genes present in MspI and HpaII digestions and Venn analyses, for each sample

Gene ontology analyses for identification of pathways enriched by the genes showing different DNA methylation status.</td><td>Comparison of genes with the same DNA methylation status among all samples and identification of shared genes and private ones
Gene ontology analyses for identification of pathways enriched for genes with the same DNA methylation status.</td><td>Differential methylation analysis among specific sets of samples</td><td></td></tr>
<tr><td colspan="2">Network analyses</td><td>BLAST analyses of contigs</td><td>BLAST analyses of contigs</td></tr>
</table>
