# Supplementary material for: Exploring the crop epigenome: a comparison of DNA methylation profiling techniques
Source: Front Plant Sci. 2023 May 30;14:1181039. doi: 10.3389/fpls.2023.1181039 (PMC10306282; doi:10.3389/fpls.2023.1181039)
Supplement: Supplementary file 2 [file DataSheet_2.pdf]

Supplementary Material

Exploring the crop epigenome: a comparison of DNA methylation profiling techniques

Dolores Rita Agius[1,2], Aliki Kapazoglou[3], Evangelia Avramidou[4], Miroslav Baranek[5], Elena Carneros[6], Elena Caro[7], Stefano Castiglione[8], Angela Cicatelli[8], Aleksandra Radanovic[9], Jean-Paul Ebejer[1], Daniel Gackowski[10], Francesco Guarino[8], Andrea Gulyás[11], Hans Hoenicka[12], Vera Inácio[13], Frank Johannes[14], Erna Karalija[15], Michal Lieberman-Lazarovich[16], Federico Martinelli[17], Stéphane Maury[18], Velimir Mladenov[19], Leonor Morais-Cecílio[20], Norbert Hidvégi[11], Aleš Pečinka[21], Eleni Tani[22], Pilar S. Testillano[6], Dimitar Todorov[23], Luis Valledor[24], Valya Vassileva[23*]

SUPPLEMENTARY DATA

Mass spectrometric quantification of epigenetic modifications in DNA

Dissolve DNA pellet in 50 µL of MilliQ-grade deionized water and mix with 50 µL of NP1 buffer (200 mM ammonium acetate, 0.2 mM $ZnCl_2$; pH 4.6). Add nuclease P1 (100 U, New England Biolabs) and tetrahydrouridine (10 mg/ml) and incubate at 37°C for 3.5 h. Subsequently, add 13 µL of 10% (v/v) NH_4OH and 12 U of Shrimp Alkaline Phosphatase (rSAP, New England Biolabs) to each sample following 2 h incubation at 37 °C. Ultrafilter all hydrolysates were prior to injection and concentrate to a final volume 10 µL. Finally, spike DNA hydrolysates with a solution of internal standard, to concentration of 50 fmol/µL of [$^{13}C_{10}$, $^{15}N_2$]-5-mdC, [D3]-5-hmdC, [$^{13}C_{10}$, $^{15}N_2$]-5-fdC, [$^{13}C_{10}$, $^{15}N_2$]-5-cadC, [$^{13}C_{10}$, $^{15}N_2$]-5-hmdU, [^{13}C, $^{15}N_2$]-2'-deoxyuridine (dU), and [$^{15}N_5$]-8-oxo-7,8-dihydro-2'-deoxyguanosine (8-oxodG). Perform chromatographic separation using a method described earlier by Starczak et al. (2022) in 2D-UPLC system with photo-diode array detector for the first dimension chromatography (used for the quantification of unmodified deoxynucleosides) and tandem quadrupole mass spectrometer using columns: Waters Cortecs T3 column (150 mm×3 mm, 1.6 µm) with a precolumn at the first dimension, a Waters X-select C18 CSH (100 mm×2.1 mm, 1.7 µm) at the second dimension and Waters X-select C18 CSH (20 mm×3 mm, 3.5 µm) as a trap/transfer column. At the first dimension use the flow rate was 0.5 mL/min, the injection volume 2 µL and gradient elution for 10 minutes using a mobile phase 0.05 % acetate (A) and acetonitrile (B) (0.7-5 % B for 5 minutes, followed by the column washing with 30 % acetonitrile and re-equilibration with 99 % A for 3.6 minutes). At the second dimension use the flow rate 0.35 mL/min. in a gradient elution for 10 minutes using a mobile phase 0.01 % acetate (A) and methanol (B) (1-50 % B for 4 minutes, isocratic flow of 50 % B for 1.5 minutes, and re-equilibration with 99 % A up to the next injection). Analyze all the samples in three to five technical replicates and use the technical mean for further calculation. Transition patterns for all the analyzed compounds along with specific detector settings are given in the Supplementary table 2.

SUPPLEMENTARY TABLE 2. Transition patterns, specific detector settings and sources of standards for analyzed compounds.

Compound name	Ionization mode	Nominal molecular mass (Da)	Pseudomolecular ion formulation	Nominal parent ion (Da)	Nominal daughter ion (Da)	ESI capillary (kV)	ESI cone (V)	Collision energy (eV)	Standard source
5-(hydroxymethyl)-2'-deoxycytidine	positive	257	$[M+H]^+$	258	124	1.2	15	10	Berry & Associates
[D_3]-5-(hydroxymethyl)-2'-deoxycytidine	positive	260	[(M+3)+H]+	261	127	1.2	15	10	Toronto Research Chemicals
5-formyl-2'-deoxycytidine	negative	255	[M-H]-	254	121	3.5	28	18	Berry & Associates
[$^{13}C_{10}$, $^{15}N_2$]-5-formyl-2'-deoxycytidine	negative	267	[(M+12)-H]-	266	128	3.5	28	18	own synthesis
5-carboxy-2'-deoxycytidine	negative	271	[M-H]-	270	110	3.5	20	20	Berry & Associates
[$^{13}C_{10}$, $^{15}N_2$]-5-carboxy-2'-deoxycytidine	negative	283	[(M+12)-H]-	282	116	3.5	20	20	own synthesis
5-(hydroxymethyl)-2'-deoxyuridine	negative	258	[M-H]-	257	124	3.5	20	15	Berry & Associates
[$^{13}C_{10}$, $^{15}N_2$]-5-(hydroxymethyl)-2'-deoxyuridine	negative	270	[(M+12)-H]-	269	131	3.5	20	15	own synthesis
2'-deoxyuridine	negative	228	[M-H]-	227	184	3.5	20	12	Sigma-Aldrich
[^{13}C, $^{15}N_2$]-2'-deoxyuridine	negative	231	[(M+3)-H]-	230	185	3.5	20	12	Medical Isotopes
8-oxo-7,8-dihydro-2'-deoxyguanosine	negative	283	[M-H]-	282	192	1.2	20	15	Sigma-Aldrich
[$^{15}N_5$]-8-oxo-7,8-dihydro-2'-deoxyguanosine	negative	288	[(M+5)-H]-	287	197	1.2	20	15	Cambridge Isotope Laboratories
5-methyl-2'-deoxycytidine	positive	241	[M+H]+	242	126	1.2	12	18	Jena Bioscience
[$^{13}C_{10}$, $^{15}N_2$]-5-methyl-2'-deoxycytidine	positive	253	[(M+12)+H]+	254	133	1.2	12	18	own synthesis
N6-methyl-2’-deoxyadenosine	positive	265	[M+H]+	266	150	3.5	15	15	Toronto Research Chemicals
[D_3]-N6-methyl-2’-deoxyadenosine	positive	268	[(M+3)+H]+	269	153	3.5	15	18	Toronto Research Chemicals
